# Clonal dynamics of *BRAF*-driven drug resistance in *EGFR*-mutant lung cancer

**DOI:** 10.1038/s41698-021-00241-9

**Published:** 2021-12-17

**Authors:** Diana Schaufler, David F. Ast, Hannah L. Tumbrink, Nima Abedpour, Lukas Maas, Ayla E. Schwäbe, Inga Spille, Stefanie Lennartz, Jana Fassunke, Mihaela Aldea, Benjamin Besse, David Planchard, Lucia Nogova, Sebastian Michels, Carsten Kobe, Thorsten Persigehl, Theresa Westphal, Sophia Koleczko, Rieke Fischer, Jan-Phillip Weber, Janine Altmüller, Roman K. Thomas, Sabine Merkelbach-Bruse, Oliver Gautschi, Laura Mezquita, Reinhard Büttner, Jürgen Wolf, Martin Peifer, Johannes Brägelmann, Matthias Scheffler, Martin L. Sos

**Affiliations:** 1 University of Cologne, Faculty of Medicine and University Hospital Cologne, Department I of Internal Medicine, Center for Integrated Oncology Aachen Bonn Cologne Duesseldorf, Network Genomic Medicine, Lung Cancer Group Cologne, Cologne, Germany; 2 University of Cologne, Faculty of Medicine and University Hospital Cologne, Institute of Pathology, Molecular Pathology, Cologne, Germany; 3 University of Cologne, Faculty of Medicine and University Hospital Cologne, Department of Translational Genomics, Cologne, Germany; 4 Mildred Scheel School of Oncology, Faculty of Medicine and University Hospital Cologne, University of Cologne, Cologne, Germany; 5 University of Cologne, Faculty of Medicine and University Hospital Cologne, Center for Molecular Medicine Cologne, Cologne, Germany; 6 University of Cologne, Faculty of Medicine and University Hospital Cologne, Institute of Pathology, Network Genomic Medicine, Cologne, Germany; 7 Department of medical oncology, Thoracic Group, Gustave Roussy, Villejuif, Paris Sud University Orsay, Paris, France; 8 University of Cologne, Faculty of Medicine and University Hospital Cologne, Department of Nuclear Medicine, Cologne, Germany; 9 University of Cologne, Faculty of Medicine and University Hospital Cologne, Institute of Diagnostic and Interventional Radiology, Cologne, Germany; 10 Cologne Center for Genomics, University of Cologne, Cologne, Germany; 11 DKFZ, German Cancer Research Center, German Cancer Consortium (DKTK), Heidelberg, Germany; 12 University of Bern and Cantonal Hospital of Lucerne, Lucerne, Switzerland; 13 Medical Oncology Department, Hospital Clinic, Laboratory of Translational Genomics and Targeted therapies in Solid Tumors, IDIBAPS, Barcelona, Spain

**Keywords:** Translational research, Non-small-cell lung cancer, Molecular medicine

## Abstract

Activation of MAPK signaling via *BRAF* mutations may limit the activity of EGFR inhibitors in *EGFR-*mutant lung cancer patients. However, the impact of *BRAF* mutations on the selection and fitness of emerging resistant clones during anti-EGFR therapy remains elusive. We tracked the evolution of subclonal mutations by whole-exome sequencing and performed clonal analyses of individual metastases during therapy. Complementary functional analyses of polyclonal *EGFR*-mutant cell pools showed a dose-dependent enrichment of *BRAF*^*V600E*^ and a loss of EGFR inhibitor susceptibility. The clones remain stable and become vulnerable to combined EGFR, RAF, and MEK inhibition. Moreover, only osimertinib/trametinib combination treatment, but not monotherapy with either of these drugs, leads to robust tumor shrinkage in *EGFR*-driven xenograft models harboring *BRAF*^*V600E*^ mutations. These data provide insights into the dynamics of clonal evolution of *EGFR*-mutant tumors and the therapeutic implications of *BRAF* co-mutations that may facilitate the development of treatment strategies to improve the prognosis of these patients.

## Introduction

Targeted treatment of epidermal growth factor receptor (*EGFR*)-mutant non-small cell lung cancer (NSCLC) is a landmark for rational therapy addressing molecular vulnerabilities^1^. Treatment with first- and second-generation EGFR tyrosine kinase inhibitors (TKIs) markedly improved the clinical outcome of patients with advanced *EGFR*-mutant NSCLC^2–5^. Currently, osimertinib is the only third-generation EGFR inhibitor approved for the sequential treatment of patients with acquired *EGFR*^*T790M*^ resistance mutation occurring after first- and second-generation TKIs^6,7^. In addition, osimertinib became the new standard-of-care in the first-line treatment of patients with *EGFR*-mutant NSCLC^8,9^.

Despite the clinical efficacy of osimertinib in the first- and second-line treatment of *EGFR*-mutant NSCLC, drug resistance with disease progression is inevitable^10–18^. Various *EGFR*-dependent and *EGFR*-independent resistance mechanisms have been identified including *EGFR*^*C797S*^ and *EGFR*^*G724S*^ mutations, *MET*/*HER2* amplification, activation of the RAS–mitogen-activated protein kinase (MAPK) or RAS–phosphatidylinositol 3-kinase (PI3K) pathways, new fusions, and histological transformation. RAS–MAPK pathway aberrations that are known to confer resistance to osimertinib include *BRAF*, *NRAS*, and *KRAS* mutations^10,19,20^. *BRAF* mutations occur in 2–4% of NSCLC patients and the vast majority are localized in the kinase domain, including the most common mutation *BRAF*^*V600E*^. *BRAF* mutations can be categorized into three classes based on their ability to act as monomers or dimers and based on their kinase activity. *BRAF*^*V600E*^ mutations represent class I mutations that, similarly to class II *BRAF* mutations (*RAS*-independent), result in activation of the BRAF kinase and the MAPK pathway (gain of function). Class III *BRAF* mutations (*RAS*-dependent) result in an impaired BRAF kinase activity and amplify ERK signaling depending on upstream activating signals (e. g. *RAS* activating mutations, *NF1* tumor suppressor deletion)^21^. All classes of *BRAF* mutations are recognized as oncogenic driver mutations, yet only *BRAF*^*V600E*^ mutations represent clinically actionable drug targets in cancer patients^22,23^.

*BRAF*^*V600E*^ mutations have been identified as a resistance mechanism to osimertinib in roughly 3% of cases with *EGFR*-mutant lung cancer, with or without concurrent *EGFR*^*T790M*^ mutation^10,19,20^. Several combination therapies have been proposed for *BRAF* resistance in *EGFR*-mutant lung cancer, but an integrated genomic analysis of these tumors is lacking and precludes an optimization of therapeutic regimen^24–27^. Furthermore, the current understanding of the clonal evolution of *EGFR*-mutant cells that concomitantly acquire *BRAF* mutations during anti-EGFR therapy remains limited.

Within the present study, we aimed for a comprehensive and translational approach to systematically characterize the role of co-occurring *EGFR*/*BRAF* mutations in patients with advanced lung adenocarcinoma.

## Results

### Targeting *BRAF*-driven resistance in *EGFR*-mutant lung cancer

**Fig. 1:**
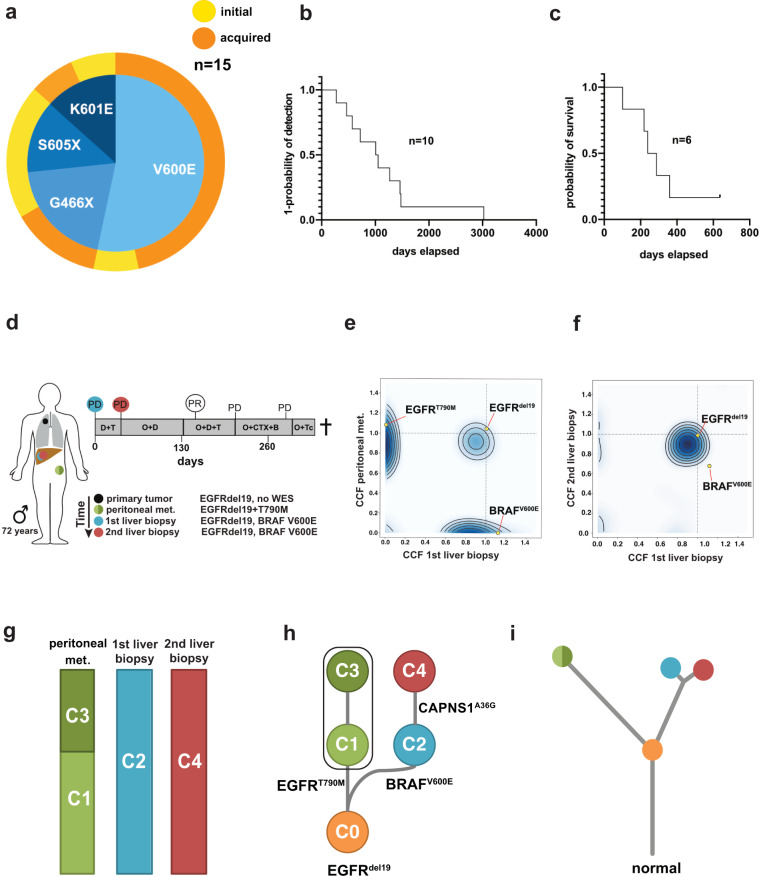
Clinicopathological characteristics for the study cohort and clonal evolution. **a** Spectrum and distribution of *BRAF* co-mutations in patients with *EGFR*-mutant lung adenocarcinoma. **b** Kaplan–Meier curve of the time elapsed from the detection of the *EGFR* mutation until the detection of the acquired *BRAF* mutation (as events) in days. **c** Kaplan–Meier curve of overall survival for patients P01, P04, P12–P15 that were available for survival analysis. **d** Overview of the biopsies and key molecular findings by NGS for patient P04. Flow chart (top right) summarizes lines of therapy approaches overtime after the acquisition of *BRAF*^*V600E*^ mutation. **e**, **f** Clustering of WES-derived mutations based on their CCFs between pairs of tumor biopsies to detect clusters of shared clonal and private mutations. Candidate mutations in *EGFR* and *BRAF* are highlighted. **g** Subclonal composition in individual biopsies indicating two subclones (C1, C3) in the peritoneal metastasis and single clones in the liver metastases. **h** Clonal evolution of reconstructed cell populations presented as a phylogenetic tree. The computationally inferred most common ancestor C0 is common to all subsequent clones and highlighted mutations are present in descendent clones. (**i**) Visualization of evolutionary genetic distances between normal tissue and longitudinal biopsies. WES whole-exome sequencing, NGS next-generation sequencing, PD progressive disease, PR partial response, D + T dabrafenib+trametinib, O + D( + T) osimertinib+dabrafenib(+trametinib), O + CTX + B osimertinib+chemotherapy+bevacizumab, O + Tc TACE osimertinib+transarterial chemoembolization, C clone, CCF cancer cell fraction.

To characterize the role of *BRAF* mutations in the context of druggable *EGFR* mutations, data of eligible patients from several centers were analyzed (see “Methods”). This led to the identification of 15 patients with lung adenocarcinoma harboring activating *EGFR* mutations and co-occurring *BRAF* mutations (Fig. 1a, Table 1). In five cases, *EGFR* and *BRAF* mutations were detected at the time of initial diagnosis, whereas in ten patients, *BRAF* mutations were acquired after anti-EGFR therapy (Table 1). In eight patients, *BRAF* mutations occurred after osimertinib treatment, in one patient after gefitinib treatment, and in one patient after afatinib treatment. The treatment history of these ten patients before the detection of acquired *BRAF* mutations is outlined in Supplementary Fig. 1. The median duration of time elapsed from diagnosis of *EGFR*-mutant lung cancer to the detection of acquired *BRAF* mutation was 33.8 months (95% CI: 9.0–99.1 months) (Fig. 1b). Six patients were evaluable for analysis of subsequent treatment and outcome after detection of acquired *BRAF* mutation (Fig. 1c, Table 2). Median overall survival (OS) for these six patients after detection of *BRAF*-driven acquired resistance was 7.8 months (95% CI: 5.1–10.5 months; Fig. 1c). Of which, four patients (P12–P15) presenting with acquired activating *BRAF*^*V600E*^ and *BRAF*^*K601E*^ (gain of function) mutations received either osimertinib and bevacizumab (*n* = 2), osimertinib and chemotherapy (*n* = 1), or chemotherapy plus bevacizumab (*n* = 1) as the next line of treatment after detection of the *BRAF* mutation (Table 2). In the Cologne cohort, we detected 26/1951 co-occurrences of *EGFR* and *BRAF* mutations (1.3%) but narrowed it down to clearly activating *EGFR* mutations. In the Paris cohort, we detected 4/184 co-occurrences of *EGFR* and *BRAF* mutations (2.2%). Overall, our data show that *BRAF* mutations represent a resistance mechanism in a relevant proportion of *EGFR*-mutant patients, warranting further investigation of the underlying clinical and evolutionary dynamics.

**Table 1:** Clinicopathological characteristics for the study cohort.

Patient ID	Sex	Age	Biopsy	*EGFR* mutation	*BRAF* mutation		Co-mutations	
01	F	70	TB	E746_A750del, T790M	V600E	Class I	Acquired (osimertinib)	Persistent T790M
02	F	71	TB	E746_A750del, T790M	S605C	Not classified	Initial	TP53 R273H
03	M	61	TB	L858R	K601E	Class II	Initial	DDR2 R279M
04	M	72	TB	E746_A750del, T790M	V600E	Class I	Acquired (osimertinib)	loss of T790M
05	M	77	TB	L861Q	G466A	Class III	Acquired (afatinib)	ERBB2 G815A, TP53 S166*
06	M	66	TB	L858R, V834L	V600E	Class I	Acquired (osimertinib)	loss of *EGFR* mutations
07	F	84	TB	L858R	V600E	Class I	Initial	–
08	F	74	TB	L858R	G466E	Class III	Acquired (gefitinib)	–
09	M	50	TB	E746_A750del	V600E	Class I	Acquired (osimertinib)	CCDC6-RET
10	F	67	TB	L747_P753delinsS	G466E	Class III	Initial	*KRAS* A59E
11	F	75	TB	E746_A750del	S605N	Not classified	Initial	–
12	F	61	LB	E746_A750del, 790M, C797S, C797G	V600E	Class I	Acquired (osimertinib)	TP53 splice
13	M	50	LB	L747_S752del	K601E	Class II	Acquired (osimertinib)	TP53 R248G
14	F	70	TB	L858R, T790M, C797S	V600E	Class I	Acquired (osimertinib)	TP53 K120E, BRCA S237Y (VUS)
15	M	52	TB	L747_A750delinsP T790M, C797G	V600E	Class I	Acquired (osimertinib)	CTNNB1 S37C, ATM R1437K (VUS)

Patients with lung adenocarcinoma harboring activating *EGFR* mutations and co-occurring *BRAF* mutations were collected from three different cancer centers. Class I and class II (RAS-independent) *BRAF* mutations result in activation of the BRAF kinase and the MAPK pathway. Class III (RAS-dependent) *BRAF* mutations result in impaired BRAF kinase activity and amplify ERK signaling based upon upstream activating signals. *BRAF*^*S605C/N*^ mutations (variants) lie within the kinase domain of the BRAF protein, they are not yet functionally classified. TB tissue biopsy, LB liquid biopsy.

**Table 2:** Systemic treatment lines and outcome evaluable for six patients after detection of the acquired *BRAF* mutation.

Patient ID	*BRAF* mutation		Time to detection of *BRAF* mutation after diagnosis (months)	Treatment after detection of *BRAF* mutation	TTD (days)	OS (days)	Outcome
01	V600E	(Class I)	96	Dabrafenib+trametinib (1 L)	74	636	Alive
				Osimertinib+dabrafenib (2 L)	27		
				Afatinib+crizotinib (3 L)	65		
				Osimertinib+dabrafenib + (4 L) trametinib	288		
				Osimertinib+bevacizumab (5 L)	53		
				Afatinib+crizotinib (6 L)	105		
				Osimertinib+dabrafenib + (7 L) trametinib	na		
04	V600E	(Class I)	47	Dabrafenib+trametinib (1 L)	38	287	Deceased
				Osimertinib+dabrafenib (2 L)	93		
				Osimertinib+dabrafenib + (3 L) trametinib	77		
				Osimertinib+carboplatin + (4 L)	75		
				Pemetrexed+bevacizumab osimertinib+TACE (5 L)	na		
12	V600E	(Class I)	38	Carboplatin+paclitaxel + (1 L) bevacizumab	68	101	Deceased
13	K601E	(Class II)	26	Osimertinib+paclitaxel (1 L)	50	239	Deceased
14	V600E	(Class I)	34	Osimertinib+bevacizumab (1 L), carboplatin+gemcitabine (2 L)	92, 40	359	Deceased
15	V600E	(Class I)	51	Osimertinib+bevacizumab (1 L) carboplatin+paclitaxel + (2 L) bevacizumab	57, 163	219	Deceased

*BRAF*^*V600E*^ and *BRAF*^*K601E*^ mutations result in an increased BRAF kinase activity. See also Fig. 1c for the Kaplan–Meier curve of OS. TTD time-to-treatment discontinuation, OS overall survival: time from acquired resistance (date of biopsy) until death/last day of follow-up, TACE transarterial chemoembolization.

Next, we selected two patients (P01, P04) who acquired *BRAF*^*V600E*^ mutation under osimertinib treatment to evaluate the safety and efficacy of various drug combinations including EGFR, RAF, MEK, or MET inhibitors, chemotherapy, or bevacizumab (Table 2, Fig. 1d, Supplementary Fig. 2a). We chose functional imaging by FDG-PET for (early) metabolic response evaluation during our investigational conduct (Supplementary Table 1) and monitored treatment-related adverse events that were predominantly of low grade and manageable (Supplementary Table 2). Osimertinib treatment was initiated and carried out for 16 months in P01 and 7 months in P04 before the detection of progressive disease. While in P01 *EGFR*^*T790M*^ mutation was sustained, in P04, we observed a loss of *EGFR*^*T790M*^ mutation. Both patients started with dabrafenib and trametinib, which in both cases did not lead to a confirmed metabolic response. Both patients underwent a rebiopsy of progressive lesions and started immediately with osimertinib and dabrafenib. In P01, the rebiopsy revealed an *EGFR*^*del19*^ mutation, loss of *EGFR*^*T790M*^, no *BRAF*^*V600E*^ mutation and an intermediate-level *MET* amplification (GCN 5.58, FISH). Subsequent doublet combinations of osimertinib plus dabrafenib and afatinib plus crizotinib showed either primary refractory disease or metabolic responses that could not be confirmed in the next scans. In contrast, the triple combination of osimertinib, dabrafenib, and trametinib led to a prolonged metabolic response and clinical benefit (Supplementary Table 1, Table 2). In P04, doublet combinations of osimertinib and dabrafenib led only to a short metabolic response not confirmed in the next scan, and with the addition of trametinib, we then observed a marked metabolic response in the primary lung tumor but not in the hepatic metastases. Rebiopsy of the hepatic lesions revealed an *EGFR*^*del19*^ mutation with T790M and C797S resistance mutations in *cis* and no *BRAF* mutation. Treatment was thus changed to osimertinib plus chemotherapy/transarterial chemoembolization due to progressive liver metastases. The patient, unfortunately, died a year after detection of *BRAF*^*V600E*^ resistance. Thus, biopsy-guided mutational profiling in conjunction with FDG-PET imaging can guide effective combination therapies to overcome resistance in these patients.

To investigate the clonal dynamics during the development of resistance, we performed whole-exome sequencing (WES) of biopsies from multiple time points and different metastatic sites obtained from P01 to P04 (Fig. 1d–i (P04), Supplementary Fig. 2 (P01)). For patient P01 WES could be performed on the primary tumor and two metastatic samples (Supplementary Fig. 2), while insufficient tissue, unfortunately, precluded analysis of the *BRAF*-mutant metastasis. Pairwise clustering based on the cancer cell fractions of the mutations (CCFs, i.e. frequency of occurrence in cancer cells after adjustment for purity, ploidy, and copy number (CN)^28^ revealed a high proportion of private mutations, while only a few mutations (e.g., *EGFR*^*del19*^) were clonal in all samples (Supplementary Fig. 2b–d). Due to the sequencing quality, an intra-biopsy heterogeneity analysis was not undertaken, but phylogenetic tree analysis between biopsies indicated a branched evolution during resistance development (Supplementary Fig. 2e). Interestingly, a common ancestor gave rise to the pleural upper lung lobe metastasis and clones subsequently developing into the pleural metastasis and the primary tumor. In accordance with this branching model, the CN profiles show shared alterations between all three available samples, but also CN segments exclusive to just one or a pair of samples (Supplementary Fig. 2f). Our data indicate early branching during tumor development and is in accordance with a scenario where resistant cells develop in parallel to the primary tumor even before treatment pressure is applied.

For patient P04 WES was performed on a peritoneal metastasis that occurred during initial inhibitor treatment (*EGFR*^*del19*^ and *EGFR*^*T790M*^), a liver metastasis 6 months after treatment had been switched to osimertinib (*EGFR*^*del19*^ and *BRAF*^*V600E*^) and a rebiopsy of the same liver lesion at progressive disease under dabrafenib and trametinib treatment (*EGFR*^*del19*^ and *BRAF*^*V600E*^) (Fig. 1d). Comparative pairwise CCF-based clustering showed only a few mutations to be shared between the peritoneal biopsy and the first liver biopsy (e.g., *EGFR*^*del19*^), while the majority was private for each one of metastases (e.g., *EGFR*^*T790M*^ and *BRAF*^*V600E*^, respectively) (Fig. 1e). In contrast, almost all mutations were found to be shared between both biopsies of the liver lesion (Fig. 1f). Subclonal composition analysis of the peritoneal metastasis revealed two subclones (C1 60%, C3 40%), while the liver metastases presented with one dominant clone each (Fig. 1g).

For subsequent phylogenetic analyses, a founder clone C0 was derived based on the mutations shared by all biopsies since the material of the primary tumor was unavailable for WES. Tracking the genomic development from C0 indicated a branched evolution diverging towards the peritoneal metastasis carrying *EGFR*^*T790M*^ with its first subclone C1, which further spawned a new subclone C3 present in the same biopsy (Fig. 1h). The liver metastasis appeared to have developed from C0 independently of the peritoneal metastasis by acquiring the *BRAF*^*V600E*^ mutation (C2). The rebiopsy of that lesion showed a distinguishable clone C4 which only carried one additional non-synonymous mutation of unknown biological relevance indicating a high degree of genetic similarity (Fig. 1h). In addition, genetic similarities between lesions were quantified to gain further insight into the clonal evolution toward therapy resistance (see Supplementary Material for further details). This analysis supports a branched evolution trajectory model with a common ancestor giving rise to the peritoneal metastasis and liver metastases (Fig. 1i). While the peritoneal metastasis and liver metastases are not closely related, only minor changes occurred between the first and second biopsy of the liver lesion (Fig. 1i). Accordingly, the CN landscape is very similar between the peritoneal metastasis and liver metastases, but almost identical between both liver biopsies (Supplementary Fig. 3). This highlights that the different metastases and resistance mechanisms (*EGFR*^*T790M*^ and *BRAF*^*V600E*^, respectively) developed independently from a common ancestral clone rather than in a linear relationship. Also, the remarkably high similarity between both liver biopsies indicates that selection pressure did not give rise to a highly distinct new subclone, potentially due to the lack of an EGFR inhibitor in the combination treatment. However, it may also be due to resistance to anti-BRAF therapy already being present in the clone C2 that propagates to C4 or may have a non-genomic basis not detectable by WES.

For a third patient (P14) we obtained a biopsy at the time of progression under osimertinib treatment (Supplementary Fig. 4a). WES of this biopsy showed the presence of several oncogenic *EGFR* mutations, namely L858R, T790M, and C797S (Supplementary Fig. 4b). All of those mutations were clonal, the T790M and C797S mutations are in agreement with previously described resistance mechanisms to EGFR inhibitors. Interestingly, we also detected a *BRAF*^*V600E*^ mutation (Supplementary Fig. 4b, Table 1). In contrast to the *EGFR* mutations, the *BRAF* mutation was subclonal and may thus indicate the branching of a newly developing subclone. This further highlights the complexity of disease resistance, which may incorporate several mechanisms of resistance development in parallel. Overall, the clonal analyses highlight that a process of branched evolution underlies resistance to targeted treatments in patients with *EGFR*-mutant tumors and may give rise to various independent resistance mechanisms.

### Resistance through the selection of *BRAF*^*V600E*^-positive clones

**Fig. 2:**
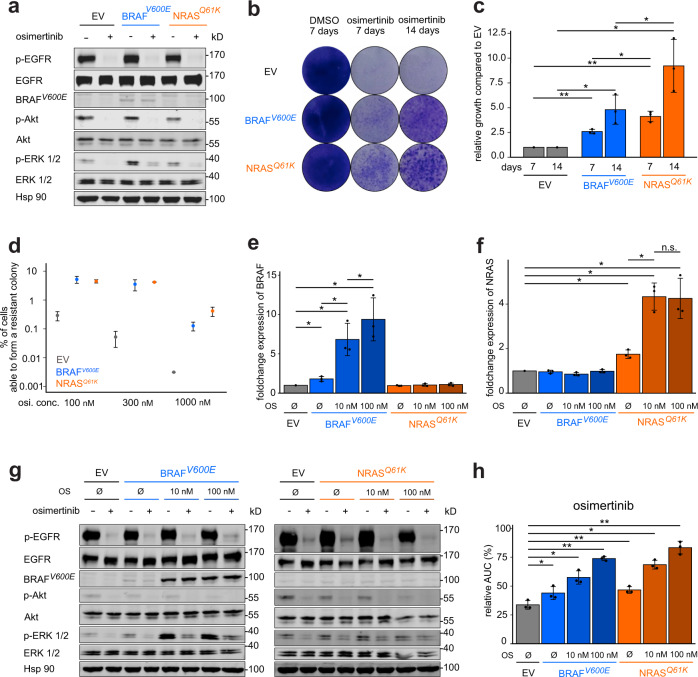
Selection of *BRAF*^*V600E*^-positive clones in *EGFR*-mutant cells. **a** Immunoblotting of PC9 cells expressing the annotated constructs, treated with (+) or without (−) osimertinib (48 h). Hsp90 is used as a loading control. **b** Clonogenicity assays of PC9 derived cell lines treated with osimertinib for 7 and 14 days or DMSO control for 7 days are displayed. **c** Quantitative analysis of (**b**) normalized to PC9 (EV). **d** Limited dilution assay of PC9-derived cell lines treated for 21 days before analysis. **e**, **f** qRT-PCR analysis of mRNA expression in **e** *BRAF* and **f** *NRAS* in PC9 derived cell lines normalized to EV. **g** Immunoblotting of PC9 cells expressing the annotated constructs that were treated as in (**a**). **h** Viability curves of PC9 cells expressing the annotated constructs treated with osimertinib (72 h) are shown. The relative area under the curve (AUC) in % compared to a theoretical non-responding AUC. Error bars indicate mean ± SD. Two-tailed paired *t* tests, ***p < 0.001, **p < 0.01, *p ≤ 0.05, ^n.s.^*p* > 0.05. *EGFR* epidermal growth factor receptor, *BRAF* B-rapidly accelerated fibrosarcoma, *NRAS* neuroblastoma rat sarcoma, EV empty vector.

To functionally validate our clinical observations, we overexpressed *BRAF*^*V600E*^ in *EGFR*^*del19*^-mutant PC9 cells. To compare *BRAF*^*V600E*^-mediated effects to upstream activation of MAPK signaling, we generated cells expressing *NRAS*^*Q61K*^, a mutation reported in preclinical models of acquired EGFR-inhibitor resistance^16,19^. In a polyclonal pool of PC9 cells stably expressing *BRAF*^*V600E*^ or *NRAS*^*Q61K*^, only modest activation of the MAPK signaling was detected as determined by immunoblotting of phospho-ERK (Fig. 2a). However, residual phospho-ERK-levels after osimertinib treatment were detected only in cells with *BRAF*^*V600E*^ or *NRAS*^*Q61K*^ overexpression but not in control PC9 empty vector (EV) cells. During 7-14 day treatment the insufficient inhibition of MAPK signaling translated into the outgrowth of osimertinib-resistant clones in cells expressing *BRAF*^*V600E*^ or *NRAS*^*Q61K*^ (Fig. 2b, c). In line with this observation, only ≤0.3% of PC9 (EV) cells were found to be able to give rise to colonies during increasing doses of osimertinib treatment (Fig. 2d). However, overexpression of *BRAF*^*V600E*^ or *NRAS*^*Q61K*^ significantly enhanced the pool of cells with the capacity to outgrow during therapy to 5.2% (*BRAF*) and 4.5% (*NRAS*) or less in a dose-dependent manner (Fig. 2d).

The next question was whether the enrichment of cells with high *BRAF*^*V600E*^ or *NRAS*^*Q61K*^ expression would have an impact on EGFR inhibitor sensitivity. Therefore, polyclonal PC9^BRAF-V600E^ and PC9^NRAS-Q61K^ cells were preselected either with 10 nM (PC9^BRAF/NRAS^ OS 10 nM) or 100 nM (PC9^BRAF/NRAS^ OS 100 nM) of osimertinib over the course of >30 days. Using RT-PCR a dose-dependent elevation of RNA levels of the respective resistance alleles was found in PC9^BRAF^ OS and PC9^NRAS^ OS cells after osimertinib selection (Fig. 2e, f). Osimertinib-preselected cells exhibited a higher induction of *BRAF*^*V600E*^ expression (9.39-fold) than *NRAS*^*Q61K*^ expression (4.25-fold, *p* = 0.036). Accordingly, untreated osimertinib-preselected cells with high *BRAF*^*V600E*^ expression displayed stronger phospho-ERK staining when compared to *NRAS*^*Q61K*^ (Fig. 2g). Both osimertinib-preselected PC9^BRAF-V600E^ and PC9^NRAS-Q61K^ cells showed higher levels of sustained phospho-ERK during osimertinib treatment (Fig. 2g) and a higher degree of resistance in viability assays compared to non-selected cells (Fig. 2h). A similar degree of resistance was observed against the EGFR inhibitors erlotinib or afatinib (Supplementary Fig. 5a, b) but not against the non-specific, chemotherapeutic cisplatin (Supplementary Fig. 5c).

To further substantiate our data in an independent model, *BRAF*^*V600E*^ was overexpressed in the *EGFR*^*del19*^-mutant HCC827 cell line. Again, a dose-dependent induction of resistance through osimertinib-preselection was observed in polyclonal HCC827^BRAF-V600E^ cell pools (Supplementary Fig. 5d). These findings are in line with our clinical observations and previous cases that identified *BRAF*-mediated resistance in *EGFR*-mutant tumors during anti-EGFR therapy. Our results suggest that *BRAF*-mutant clones are enriched through EGFR-directed therapy in *EGFR*-mutant adenocarcinoma.

### Overcoming *BRAF*^*V600E*^-mediated resistance in *EGFR*-mutant cells

**Fig. 3:**
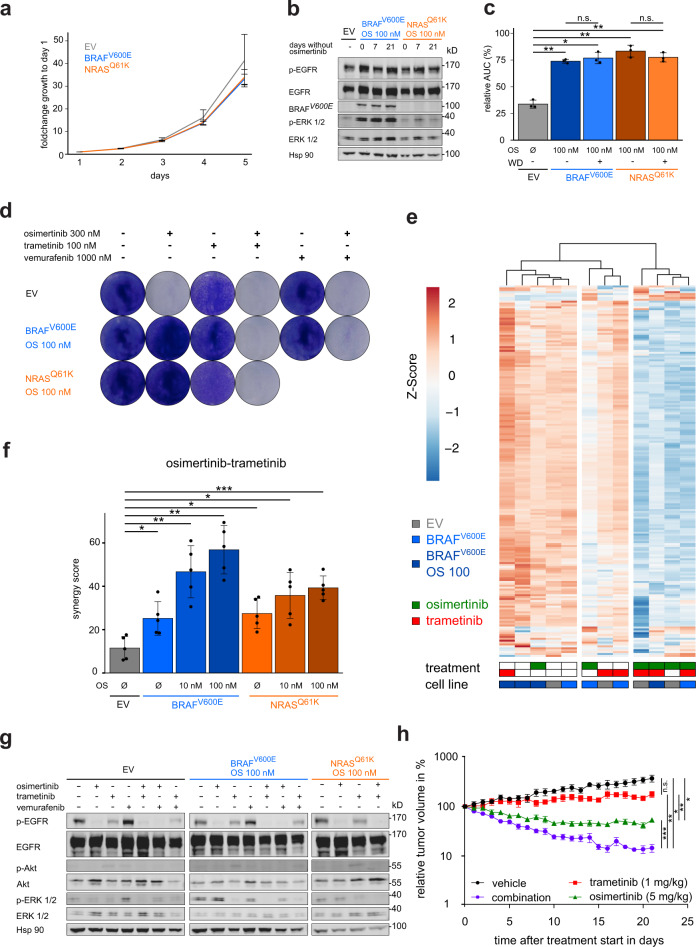
Overcoming *BRAF*^*V600E*^-mediated resistance in *EGFR*-mutant cells. **a** Growth series of PC9 derived cell lines counted for 5 days every 24 h (see Methods). **b** Immunoblotting of PC9^BRAF-V600E^ OS 100 nM, PC9^NRAS-Q61K^ OS 100 nM, and PC9 (EV). Osimertinib-preselected cells were cultured for 0, 7, and 21 days without osimertinib treatment and plated 48 h before lysis. **c** Cell viability assay of PC9 cells expressing the annotated constructs treated for 72 h with osimertinib is shown. The relative AUC (see Methods) of BRAF^V600E^ OS 100 nM and NRAS^Q61K^ OS 100 nM after osimertinib withdrawal for >40 days are shown. **d** Clonogenicity assay of PC9 cells expressing the annotated constructs treated for 14 days with indicated compounds before staining. **e** RNA-seq based expression of E2F gene set genes (rows) in PC9 derived cell lines (columns) after 48 h treatment with indicated inhibitors. Expression was normalized as *z*-score per gene. **f** Synergy screen of osimertinib and trametinib combination treatment in PC9 derived cell lines for 72 h are displayed. **g** Immunoblotting of PC9 cells expressing the annotated constructs is shown. Treatment with indicated compounds 48 h before lysis. **h** Relative tumor volume of xenograft mice injected with PC9^BRAF-V600E^ OS 100 nM cells in % compared to day 0 of the treatment regimen (see Methods). Error bars indicate mean ± SD. Two-tailed paired *t* tests (all except (**h**); two-tailed Welch’s *t* tests with Bonferonni-correction), ***p < 0.001, **p < 0.01, *p < 0.05, ^n.s.^*p* > 0.05. *EGFR* epidermal growth factor receptor, *BRAF* B-rapidly accelerated fibrosarcoma, *NRAS* neuroblastoma rat sarcoma, EV empty vector.

Previous studies have found that concomitant *KRAS* and *EGFR* mutations may increase the cell death rate of adenocarcinoma cells through hyperactivation of ERK signaling^29,30^. We tested whether the activation of MAPK signaling via *BRAF*^*V600E*^ may have a similar effect in *EGFR*-mutant PC9 cells. To this end, the cell proliferation of PC9^BRAF-V600E^ and PC9^NRAS-Q61K^ was measured over 5 days, but no major differences were observed compared to EV cells (Fig. 3a). We also did not detect any differences in the basal cell death rate between cell lines (Supplementary Fig. 6a). Consequently, cells with high expression of mutant *BRAF*/*NRAS* did not get counter-selected after the withdrawal of osimertinib (Fig. 3b, c).

Next, we tested combination therapies by targeting EGFR and MAPK signaling individually in PC9^BRAF-V600E^ and PC9^NRAS-Q61K^ cells (Fig. 3d). Both MEK inhibition and BRAF inhibition, as monotherapy, had a limited effect on the viability of PC9^BRAF-V600E^ mutant cells (Supplementary Fig. 6b, c). In contrast, the combination of osimertinib and MEK or BRAF inhibition effectively prevented the outgrowth of colonies (Fig. 3d). To further validate our previous findings on a transcriptional level, we performed RNA sequencing of PC9 (EV), PC9^BRAF-V600E^, and PC9^BRAF-V600E^ OS 100 nM cells treated with osimertinib, trametinib, a combination of both or control for 48 h (see Supplementary Material). As expected, a principal component analysis showed that osimertinib monotherapy had strong effects only on PC9^BRAF-V600E^ cells, while trametinib plus osimertinib comparably impacted both PC9^BRAF-V600E^ and PC9^BRAF-V600E^ OS 100 nM cells (Supplementary Fig. 6d). We next clustered samples based on the expression of E2F target genes to assess the impact on cell cycle-related gene expression as a surrogate marker for the cytotoxic effects of the given perturbation (Fig. 3e). In this analysis, the strongest downregulation of E2F genes was present in the group of cell line/treatment combinations that led to reduced cell numbers in crystal violet assays (Fig. 3d). Repression of E2F target genes was lower in unselected PC9^BRAF-V600E^ cells with osimertinib compared to PC9 EV cells or compared to combination treatment (Fig. 3d). This indicates the limited efficacy of osimertinib monotherapy treatment if a *BRAF* mutation is present even without prior selection and supports the use of combination treatment. Furthermore, in PC9^BRAF-V600E^ OS 100 nM cells the expression of MAPK pathway responsive genes was only perturbed during osimertinib and trametinib treatment (Supplementary Fig. 7a)^31^. Next, we assessed the synergy between osimertinib and trametinib, using ZIP-based synergy analysis, and found a strong synergy that correlated with the expression of BRAF-V600E in PC9 cells (Fig. 3f, see Methods). The calculated synergy score for osimertinib and vemurafenib was limited and we found an antagonism for the combination of trametinib and vemurafenib inhibition in PC9^BRAF-V600E^ mutant cells (Supplementary Fig. 7b, c). Finally, using a three-fold titration matrix, we observed a considerably low synergy for osimertinib and vemurafenib treatment compared to osimertinib and trametinib treatment, which was not further increased in a triple combination by adding vemurafenib (Supplementary Fig. 7d). In accordance with the synergy results, osimertinib with trametinib in contrast to osimertinib alone resulted in full inhibition of phospho-ERK signaling. Vemurafenib did not fully abrogate the sustained phospho-ERK signaling, as it also hyperactivated phospho-ERK as monotherapy, most likely due to the paradoxical effect on the endogenous wild type BRAF kinase (Fig. 3g)^32^. To further validate our in vitro results, we performed an in vivo study with xenografts implanted with PC9^BRAF-V600E^ cells that were preselected for high *BRAF*^*V600E*^ expression. Once the mice developed tumors, we started with the treatment regimen consisting of vehicle, osimertinib, trametinib or the combination of osimertinib and trametinib for 21 days (Fig. 3h). Compared to vehicle treatment, trametinib did not significantly decrease tumor volume, and osimertinib monotherapy led to a measurable tumor growth reduction. However, only, combination therapy led to robust tumor shrinkage in these xenografts (Fig. 3h). Thus, our in vivo data largely reflects our in vitro findings and suggests that combination therapy is necessary to induce substantial tumor shrinkage in tumors harboring activating *EGFR* and *BRAF* mutations. Of importance, none of the mice in the individual treatment arms experienced weight loss (Supplementary Fig. 7e) or any other severe treatment-associated side effects.

## Discussion

Osimertinib replaced other EGFR inhibitors in the early lines of therapy. This development had a major impact on the resistance profiles and development of effective salvage therapies^10–15,18,33^. The activation of MAPK signaling seems to play a more prominent role in patients' progressive on third-generation EGFR inhibitors when compared to first- and second-generation EGFR inhibitors^11,16,19,20,34^. Our comprehensive genomics study of *EGFR*-mutant patients with co-occurring *BRAF* mutations provides insights into the evolution of MAPK-driven resistance and its impact on *EGFR*-directed treatment.

Our combination of longitudinal clinical and genomic analyses provides insight into the subclonal heterogeneity of the individual tumors and corresponding metastases during resistance evolution. Our clonality analyses revealed that resistance to osimertinib (initiated at detection of *EGFR*^*T790M*^ mutation) and subsequent combination of dabrafenib plus trametinib (initiated at detection of *BRAF*^*V600E*^ mutation) was driven by an evolutionary branching process rather than a linear trajectory of one clone that continues to acquire additional resistance mutations. Moreover, in both patients, the different metastases are genetically distinct from each other but arise from common ancestors that do not carry a resistance mutation. Even within our limited cohort, we observe different patterns of clonal evolution: while for P04 a common ancestor most likely from the primary tumor gave rise to the different metastases, for patient P01 phylogenetic analyses are in accordance with a model supporting much earlier branching. Of note, resistance mutations such as *EGFR*^*T790M*^ and *BRAF*^*V600E*^ were not detected by either panel sequencing or WES in samples still sensitive to the respective inhibitors. This may indicate that they developed either de novo during treatment or were pre-existent, but at a frequency to low to be detected without selection pressure.

Overall, our results demonstrate the presence and further development of tumor heterogeneity that can give rise to multiple resistance mechanisms due to treatment selection pressure. Moreover, our genomic analysis emphasizes that we are faced with a complex mutational landscape based on intra-tumoral, inter-tumoral, and inter-patient heterogeneity. It thus constitutes a major clinical challenge for the development of an efficient treatment strategy to counteract tumor progression. Based on the present findings a diagnostic strategy aiming to address the multilayered heterogeneity e.g. using liquid biopsies or multiple re-biopsies appears warranted to optimize treatment schedules. Our data suggest that one promising treatment strategy for patients with concurrent *EGFR* and MAPK pathway activation may require alternating treatment regimens with intermittent changes between drug combinations based upon observed heterogenic tumor response and emerging resistance patterns. To facilitate this strategy FDG-PET can be quite useful for rapid treatment evaluation and hence, dynamic clinical management as demonstrated by our investigational approach. However, we are aware that more patients need to be profiled in the future to compliment our results.

We and others have previously found that acquired resistance through activation of MAPK signaling via *KRAS* mutations can be detected in patients receiving third-generation EGFR inhibitors^11,12^. *BRAF* mutations and *BRAF* rearrangements have also been shown to play a similar role like *KRAS* in the resistance setting of *EGFR*-mutant adenocarcinoma^16,19,34^. This is surprising as previous functional analyses indicated that mutant *KRAS* mutations may augment the cell death rate of *EGFR*-mutant cells and thereby limit the outgrowth of resistant clones^29^. Our cell line models indicate that concomitant MAPK pathway signaling is tolerated when *BRAF* or *NRAS* are activated. This corresponds with our clinical observation that *BRAF* mutations can co-occur with *EGFR* mutations even before anti-EGFR therapy. Interestingly, the levels of phospho-ERK activation differ strongly between *BRAF*- and *NRAS*-mutant cells but we did not observe major differences in the ability of these alleles to promote resistance or cell death in *EGFR*-mutant cells. These functional observations are also in line with our finding that *BRAF*/*EGFR*-mutant lung tumors are recurrently found across different cancer centers, indicating a basis for the co-existence of *BRAF*/*EGFR* mutations without selection pressure. Future studies are required to fully decipher the potential differences between MAPK signaling activation at different levels of the pathway in the context of *EGFR*-mutant lung adenocarcinoma. Nevertheless, our in vitro and in vivo findings fully support the notion that EGFR/MEK combination might be a viable option to overcome *BRAF*-driven resistance in patients with *EGFR*-mutant lung adenocarcinoma.

In summary, our data uncover basic principles of drug-induced evolutionary paths underlying *BRAF*-driven resistance in patients with lung adenocarcinoma. The integrated analyses support a model in which concomitant activation of EGFR and BRAF is selected through anti-EGFR therapy that combines well with EGFR, BRAF, and MEK inhibitors to overcome resistance. Our systematic exploration of clinically relevant drug combinations may offer additional avenues for follow-up investigations into novel targeted treatment strategies for patients with co-occurring *EGFR* and *BRAF* mutations.

## Methods

### Patients

We compiled a cohort of 15 patients with lung adenocarcinoma and activating *EGFR* mutations that harbored co-occurring *BRAF* mutations with and without prior anti-EGFR treatment. Patients were identified within the Network Genomic Medicine (NGM) Lung Cancer in Cologne, Germany, Institute Gustave Roussy in Paris, France, and Cantonal Hospital of Lucerne, Switzerland. Treatment, genetic findings, and survival of these patients were evaluated. All patients consented to be analyzed. The study was conducted in concordance with local ethical guidelines and was reviewed by the institutional ethics committee. Selected patients were treated with different lines of therapy including combinations of osimertinib, dabrafenib, and trametinib. These patients provided written informed consent for a prospective investigational molecular- and imaging-guided personalized treatment approach. Rebiopsies were acquired at disease progression. Tissue biopsy was performed through core needle biopsy according to local standard procedures. Survival of all patients was calculated using the Kaplan Meier method.

### Molecular analyses

The vast majority of the specimens analyzed in our study consisted of tumor tissue (*n* = 13). For two patients, liquid biopsies were evaluated. (Table 1). Next-generation sequencing (NGS)-based molecular profiling was performed for each patient either on tumor tissue or on circulating tumor DNA (ctDNA). For patients, P01 and P04 whole-exome sequencing was additionally performed on the tumor tissue. For patients P01 and P04, we obtained longitudinally serial repeated tissue biopsies of the leading tumor lesions at each time of progression during treatment with different combinations of osimertinib, dabrafenib and trametinib, and other therapies. NGS of tumor tissue was performed as previously described^35–38^. Plasma analysis of ctDNA was performed as previously reported^39^.

### PET-CT assessments

The efficacy of treatment was evaluated by positron emission tomography (PET)/computed tomography (CT) scans using radiolabeled ^18^F-2-fluoro-2-deoxy-d-glucose (FDG). Scans were acquired at baseline, and as early as 2 weeks (early assessment) and again at regular intervals roughly every 6 or more weeks (late assessments) after initiation or change of therapy to capture early metabolic response (measured by standard uptake value (SUV)max) and morphologic response over time. Scans were conducted as previously described and performed on a Biograph mCT Flow-Edge 128 PET/CT-system (Siemens Medical Solutions) with a 128-slice spiral CT component from the base of the skull to the mid-thigh^40^. We followed Positron Emission Response Criteria in Solid Tumors version 1.0 guidelines, assuming that response is characterized by an SUV reduction of at least 30% in the hottest lesion^41^.

### Whole-exome sequencing (WES)

WES was performed on FFPE-derived DNA from serial tumor tissue rebiopsies obtained at the time of tumor progression during treatment of patients P01 and P04. In addition, for one patient DNA was extracted from the primary tumor using the truXTRAC FFPE DNA extraction kit (Covaris, USA, Cat. No. 520307). Exomes were individually prepared using 200 ng of DNA using standard protocol SureSelectXT Automated Target Enrichment for Illumina Paired-End Multiplexed Sequencing and Agilent Bravo automated liquid handling platform. As for patient P14, there was only a post-osimertinib tumor biopsy available for WES, which was enriched using the Agilent SureSelect CR kit (Agilent, USA). After validation (2200 TapeStation, Agilent Technologies) and quantification (Qubit System, Invitrogen, Waltham, USA) pools of libraries were generated. The pools were quantified using the KAPA Library Quantification kit (Peqlab, Germany, KAPBKK4854) and 7900HT Sequence Detection System (Applied Biosystems, Waltham, USA) and subsequently sequenced at 140× mean coverage on an Illumina NovaSeq6000 sequencing instrument using a paired-end 2 × 100 bp protocol.

### WES and clonality analysis

Analysis of raw sequencing data and clonality analyses were performed using an established pipeline^42^: After alignment of the raw sequencing data to the hg19 reference genome in total 137–325 million reads could successfully be mapped per sample corresponding to a mean coverage of 93×–200× per sample and covering all intended exonic target region with ≥20× coverage for 90–98% of those. In summary, of the 42.3 megabases of exonic regions as defined by the GRCh 37/hg19 RefSeq genome annotation, 39–40 megabases of exons were sufficiently covered for mutation calling and subsequent analyses. Thus, allelic fractions of somatic mutations were corrected for purity and CN changes to determine cancer cell fractions (CCF). The distribution of CCFs was then searched for distinct subpopulations by using a nonparametric method to deconvolute the noise in the CCFs. This allows for the identification of genetically distinct tumor subclones and the reconstruction of tumor evolutionary histories.

### Cell culture and functional analyses

Human NSCLC cell lines were verified by STR profiling at the Institute for Forensic Medicine of the University Hospital of Cologne. PC9, HCC827, and HEK293T cell lines were obtained from ATCC. PC9 and HCC827 cells and their osimertinib-preselected derivatives were cultured in RPMI (Fisher Scientific, USA, Cat. No. 12004997) HEK293T cells were cultured in DMEM (Fisher Scientific, USA, Cat. No. 61965-026). All media were supplemented with 10% fetal bovine serum (Fisher Scientific, USA, Cat. No. 10270-106) and 1% penicillin/streptomycin (Fisher Scientific, USA, Cat. No. 15070-063). All cells were grown at 37 °C in a humidified atmosphere with 5% CO_2_.

### Reagents

For cell culture studies, osimertinib (LC Laboratories, USA, Cat. No. 1421373-65-0), trametinib (LC Laboratories, USA, Cat. No. 871700-17-3), and vemurafenib (LC Laboratories, USA, Cat. No. 918504-65-1) were dissolved in dimethyl sulfoxide (DMSO) (Carl Roth, Germany, Cat. No. 4720.4) to a final stock concentration of 10 mM. Cisplatin (pharmacy of University Hospital of Cologne) was diluted to 3.33 mM in 0.9% NaCl.

### Crystal violet assay

Totally, 10^5^ cells were plated into one well of a 6-well plate and treated with DMSO (control), 300 nM osimertinib, 100 nM trametinib, 1 µM vemurafenib, and combinations osimertinib plus trametinib and osimertinib plus vemurafenib. Seven or 14 days after treatment, cells were fixed in 4% paraformaldehyde (Carl Roth, Germany, Cat. No. CP10.1) in phosphate-buffered saline (PBS) (Fisher Scientific, USA, Cat. No. 14190144), stained with 0.1% crystal violet (Sigma Aldrich, USA, Cat. No. C3886-25G) in PBS, and rinsed in PBS before image acquisition. For quantification, the Crystal Violet dye was dissolved in 2 ml methanol (Carl Roth, Germany, Cat. No. CP43.4) in the 6-well plate. Twenty microlitres of this solution were diluted 1:10 with methanol and injected into 96-well plates. The read-out was the absorption at 560 nm wavelength. The results per cell line were normalized against their DMSO-controls and then against the empty vector (EV) control cell line.

### Protein overexpression experiments

Vectors pBABE puro, pBABE-puro-BRAF^V600E^, and pBABE-NRAS^Q61K^ were cotransfected with a helper plasmid into HEK 293T cells using TransIT-LT1 reagent (Mirus, USA, Cat. No. MIR2300). Forty-eight hour post transfection, replication-incompetent retroviruses were collected from the supernatant for infection of PC9 and HCC827 in the presence of 8 μg/ml polybrene (Merck Millipore, USA, Cat. No. TR1003-G). Twenty-four hour after infection, the growth medium was changed and 3 μg/ml (PC9) or 2 μg/ml (HCC827) puromycin (Sigma Aldrich, USA, Cat. No. p8833) was added for selection for 7 days. After selection, cells were analyzed for protein expression.

pBABE-puro was a gift from Hartmut Land & Jay Morgenstern & Bob Weinberg (Addgene plasmid # 1764; RRID:Addgene_1764).

pBabe-Puro-BRAF^V600E^ was a gift from William Hahn (Addgene plasmid # 15269; RRID:Addgene_15269).

pBabe-NRAS^Q61K^ was a gift from Channing Der (Addgene plasmid # 12543; RRID:Addgene_12543).

### Cell viability screening

To assess cell viability, cells were plated in 96-well plates in triplicates, and compounds were added at 9 decreasing compound concentrations 24 h after seeding. Seventy-two hours later, cell viability was measured via Cell Titer-Glo (CTG) assay (Promega, USA, Cat. No. g7573) and was normalized to DMSO-treated controls. Resistance in % was calculated as the area under the curve (AUC), calculated via Gauss’s trapezoid area formula and then divided by a theoretical non-responding AUC, all calculated in R. Data are represented as mean ± standard error of the mean and significance was calculated by paired Student’s *t* tests.

### RNA isolation and qRT-PCR

Totally, 5 × 10^5^ cells were plated into one well of a 6-well plate and harvested after 24 h. Total RNA was isolated using the RNeasy-kit (Qiagen, Germany, Cat. No. 74106) according to the manufacturer’s instructions, including DNAse I digestion (Qiagen, Germany, Cat. No. 79256). In all, 1.5 μg of total RNA was reverse transcribed using Super-script II (Thermo Fisher Scientific, USA, Cat. No. 18064022) with random hexamer primers. Quantitative real-time PCR (qPCR) was performed using the QuantStudio 3 Real-Time PCR System (Thermo Fisher Scientific) and Power SYBR Green PCR Master Mix (Thermo Fisher Scientific, USA, Cat. No. 4309155). Data were normalized to GAPDH RNA levels and are presented as mean ± SD and significance was calculated by paired Student’s *t* tests.

### Flow cytometry

Cell lines were seeded into 6-well plates (1 × 10^5^ cells/well). Twenty-four-hour later Staurosporine (Sigma Aldrich, USA, S4400) or DMSO control was added to the medium. Twenty-four-hour later supernatant was collected, cells were trypsinized (Fisher Scientific, USA, Cat. No. 11560626), washed with ice-cold PBS, and resuspended in antibody-binding buffer (10 mM HEPES pH 7.4 (Fisher Scientific, USA, Cat. No. 15630080), 140 mM NaCl; 2.5 mM CaCl_2_). Cells were stained for Annexin-V (BD Biosciences, USA, Cat. No. 556420) and 50 µg/mL propidium iodide (Carl Roth, Germany, Cat. No. CN74). After 20 min of incubation in the dark, samples were analyzed on a FACS Gallios Flow Cytometer (Beckman Coulter). We used FACS Kaluza software (Beckman Coulter) to quantify populations. At least 5 × 10^4^ events were assessed per measurement. All measurements were performed as duplicates. Gates used can be found in Supplementary Fig. 8. Data are presented as mean ± SD.

### Immunoblot

Cell lysates were prepared using RIPA buffer supplemented with protease inhibitors (Complete Mini Protease Inhibitor Cocktail, Roche, Switzerland, Cat. No. 11836170001). Protein concentration was determined by BCA assay (Thermo Fisher Scientific, USA, Cat. No. 23225) and equal amounts of protein (20 µg) were separated on 4–12% Tris-glycine sodium dodecyl sulfate-polyacrylamide gel electrophoresis gels (Thermo Fisher Scientific, USA, Cat. No. XP04125BOX) and transferred to PVDF-FL membrane (Sigma Aldrich, USA, Cat. No. IPFL00010). Membranes were blocked in 5% milk (Carl Roth, Germany, Cat. No. T145.1) blocking buffer in Tris-buffered saline (TBS), incubated with primary antibodies, washed, and incubated with fluorescently labeled secondary antibodies before detection with Odyssey CLx imaging system (LI-COR Biosciences). Images were processed using the Image Studio Software (LI-COR Biosciences). Primary antibodies are EGFR (Cell Signaling, USA, Cat. No. CS-4267), p-EGFR (Cell Signaling, USA, Cat. No. CS-3777), BRAF-V600E (Spring Bioscience, USA, Cat. No. E-19290), BRAF (Santa Cruz Biotechnology, USA, Cat. No. SC-5284), ERK (Cell Signaling, USA, Cat. No. CS-9102), p-ERK (Cell Signaling, USA, Cat. No. CS-4370), Akt (Cell Signaling, USA, Cat. No. CS-2920), p-Akt (Cell Signaling, USA, Cat. No. CS-9271) and Hsp90 (Cell Signaling, USA, Cat. No. CS-4877). All primary antibodies were diluted 1:1000 in 5% milk blocking buffer in TBS with 0.2% Tween^®^20 (Sigma Aldrich, USA, Cat. No. P7949-500ML). Secondary antibodies are goat anti-rabbit 800CW (LI-COR Biosciences, USA, Cat. No. 926-32211), goat anti-mouse 800CW (LI-COR Biosciences, USA, Cat. No. 926-3220), goat anti-rabbit 680LT (LI-COR Biosciences, USA, Cat. No. #926-68021), and goat anti-mouse 680LT (LI-COR Biosciences, USA, Cat. No. 926-68020). All secondary antibodies were diluted 1:20,000 in 2.5% milk blocking buffer in TBS with 0.2% Tween^®^20 and 0.01% sodium dodecyl sulfate (SDS) (Carl Roth, Germany, Cat. No. 8029.4).

All blots derive from the same experiment and were processed in parallel. Uncropped blots can be found in Supplementary Figs. 9–13.

### Synergy screen

Cells were plated in a 6 × 6 wells matrix in 96-well plates. After 24 h cells were treated with five decreasing concentrations of compound A plus DMSO control starting from right to left. Cells were also treated at the same time with five decreasing concentrations of compound B plus DMSO control starting from the bottom to the top. The topmost left well is only treated with DMSO, while the bottommost right well is treated with the highest concentration of both compounds. The following starting concentrations were used: 300 nM of osimertinib, 100 nM of trametinib, and 1 µM of vemurafenib. Seventy-two hours after treatment, cell viability was measured via CTG assay and was normalized to DMSO-treated controls. Synergy scores were then calculated in R using the SynergyFinder Package and the Zero Interaction Potency (ZIP) reference model as implemented in the package. The mean of the nine highest synergy scores from each matrix is presented ±SD and significance were calculated by paired Student’s *t* tests.

### 3D Synergy screen

Cells were plated as described in “Synergy screen”, just on six plates, each with a set concentration of vemurafenib to add a third dimension. Cells were treated for the same time and with the same concentration as in “Synergy screen”. Cell viability was measured the same way as in “Synergy screen”. The expected drug combination responses were calculated based on ZIP reference model using SynergyFinder^43^. Deviations between observed and expected responses with positive and negative values denote synergy and antagonism, respectively.

### Growth series

Totally, 1 × 10^5^ cells per well were plated 5 times in triplicates per cell line in 6-well plates. For 5 days, always after 24 h, one triplicate of each cell line was trypsinized and counted via Z Series Coulter Counter (Beckmann Coulter). Results were normalized to day 1 and were anticipated from the slope of a best-fitting line through each data set Data are presented as mean ± SD.

### Generating osimertinib selected cell lines

PC9^BRAF-V600E^, PC9^NRAS-Q61K^, and HCC827^BRAF-V600E^ cell lines were each treated with 10 nM or 100 nM osimertinib respectively for >30 days. After that cells were labeled osimertinib selected (OS) 10 nM or 100 nM, respectively, and experiments were performed. Even after >30 days osimertinib treatment in cell culture, cells were continuously kept under osimertinib treatment.

### 3′UTR-RNA sequencing

For each cell line (PC9 pBABE EV, PC9 pBABE BRAF^V600E^, and PC9 pBABE BRAF^V600E^ OS 100) 5 × 10^5^ cells were plated and left to adhere overnight. The next day they were treated with 300 nM osimertinib, 100 nM trametinib, a combination of both, or DMSO control for 48 h. RNA extraction and sequencing were performed using the Qiagen RNeasy Mini kit following the manufacturer’s instruction. Totally, 500 ng total RNA were used to prepare 3′ UTR mRNA libraries using the Lexogen QuantSeq kit (Lexogen, Austria, Cat. No. 015.96) according to the standard protocol^44^. Quality controlled cDNA pools were quantified with the KAPA Library Quantification kit and sequenced on a NovaSeq sequencer (Illumina, USA) with a 1 × 100 bp protocol. Raw data were aligned to the human genome reference GRCh38 using STAR aligner^45^ and gene expression was quantified with RSEM^46^ prior to downstream analysis with the R package DESeq2^47^. E2F target genes were obtained from the MSigDB Hallmark collection and MAPK feedback genes from a recently published MAPK activity score^31^.

### In vivo xenograft model

The local authorities and the animal protection committee approved all animal procedures of this study.

PC9^BRAF-V600E^ OS100 nM cells (5 × 10^6^) were resuspended in 100 µL PBS and then inoculated subcutaneously in both flanks of 8- to 12-week-old female nude mice (RJ:NMRI-FOXN1 NU, Janvier Labs) and treatment was initiated when tumors reached a mean volume of approximately 50 mm^3^. Mice were treated daily for 21 days orally with vehicle solution (1% DMSO, 30% PEG300, 0.5% hydroxypropyl methylcellulose, 0.2% Tween-80, ddH_2_O) QD, osimertinib (5 mg/kg in 1% DMSO + 30% PEG300 + ddH_2_O) QD, trametinib (1 mg/kg in 0.5% hydroxypropyl methylcellulose, 0.2% Tween-80, ddH_2_O) QD or combination (osimertinib as described before and trametinib as described before) Tumor volumes were measured daily in two dimensions using a caliper, and the volume was expressed in mm^3^ using the formula: *V* = 0.5 × (length × width^2^), where *V* is tumor volume, length is the longest tumor dimension and width is the longest tumor dimension perpendicular to the length.

### Reporting summary

Further information on research design is available in the Nature Research Reporting Summary linked to this article.

## Supplementary information

Supplementary Information

Reporting Summary

## Data Availability

The data generated and analyzed during this study are described in the manuscript and Supplementary Material. WES data were uploaded to The European Genome-phenome Archive (EGA) with the accession code EGAS00001005614. RNA sequencing data were uploaded to ArrayExpress with the accession code E-MTAB-11004. All relevant data and materials that support the findings of this work are available from the corresponding authors upon reasonable request.

## Code availability

No custom code or algorithms were created.
