# Supplementary material for: Clonal dynamics of BRAF-driven drug resistance in EGFR-mutant lung cancer
Source: NPJ Precis Oncol. 2021 Dec 17;5:102. doi: 10.1038/s41698-021-00241-9 (PMC8683498; doi:10.1038/s41698-021-00241-9)
Supplement: Supplementary file 1 — Supplementary Information [file 41698_2021_241_MOESM1_ESM.pdf]

Supplementary Material

Supplementary Table 1 (A-D) ^{18}FDG-PET/CT assessment for monitoring of metabolic response during different lines of treatment of two patients with *EGFR*/*BRAF*-mutant lung adenocarcinoma. Table summarizes the standard uptake values (SUV). **(A)** P01, lesion 1; **(B)** P01, lesion 2; **(C)** P04, lesion 1; **(D)** P04, lesion 2. D+T, dabrafenib+trametinib; O+D(+T), osimertinib+dabrafenib(+trametinib);O(+CTX)+B,osimertinib(+chemotherapy)+bevacizumab; A+C, afatinib+crizotinib; O+TACE, osimertinib+transarterial chemoembolization; FU, Follow-up; PD, progressive disease.

A

Treatment	^{18}FDG-PET/CT assessments		Left upper lung lobe (initially hottest lesion) SUVmax
D+T	BASELINE	28.11.2018	11,64
D+T	2 WeFU	19.12.2018	9,49
D+T	6 WeFU	16.01.2019	10,64
D+T	10 WeFU	18.02.2019	11,89
O+D	2 WeFU	13.03.2019	14,46
A+C	2 WeFU	09.04.2019	8,12
A+C	6 WeFU	08.05.2019	10,91
O+D+T	3 WeFU	26.06.2019	9,83
O+D+T	7 WeFU	29.07.2019	11,89
O+D+T	12 WeFU	12.09.2019	7,74
O+D+T	20 WeFU	12.11.2019	8,07
O+D+T	32 WeFU	13.02.2020 (only CT)	Morphological PD
O+D+T	9 MoFU	20.03.2019	7,13
O+Beva (2x)	6 WeFU	06.05.2020	11,96
A+C	3 WeFU	09.06.2020	8,83
A+C	12 WeFU	18.08.2020	8,68 new PET positive retroperitoneal metastases
O+D+T	not done	not done	not done

B

Treatment	^{18}FDG-PET/CT assessments		Left lower lung lobe SUVmax
D+T	BASELINE	28.11.2018	10,36
D+T	2 WeFU	19.12.2018	6,29
D+T	6 WeFU	16.01.2019	8,20
D+T	10 WeFU	18.02.2019	8,24
O+D	2 WeFU	13.03.2019	11,01
A+C	2 WeFU	09.04.2019	6,24
A+C	6 WeFU	08.05.2019	10,84
O+D+T	3 WeFU	26.06.2019	9,39
O+D+T	7 WeFU	29.07.2019	7,74
O+D+T	12 WeFU	12.09.2019	5,24
O+D+T	20 WeFU	12.11.2019	6,95
O+D+T	32 WeFU	13.02.2020 (only CT)	Morphological PD
O+D+T	9 MoFU	20.03.2019	6,44
O+Beva (2x)	6 WeFU	06.05.2020	10,01
A+C	3 WeFU	09.06.2020	7,52
A+C	12 WeFU	18.08.2020	5,53<br>new PET positive retroperitoneal metastases
O+D+T	not done	not done	not done

C

Treatment	^{18}FDG-PET/CT assessments		Right upper lung lobe (hottest lesion) SUVmax
D+T	BASELINE	13.11.2018	8,41
D+T	2 WeFU	21.12.2018	12,25
D+T	6 WeFU	08.01.2019	10,99
O+D	2 WeFU	28.01.2019	9,05
O+D	6 WeFU	27.02.2019	10,89
O+D	12 WeFU	10.04.2019	10,56
O+D+T	2 WeFU	20.05.2019	5,91
O+D+T	6 WeFU	21.06.2019	10,46
O+CTX+B (2x)	4 WeFU	22.08.2019	6.37
O+TACE	not done	not done	not done

D

Treatment	^{18}FDG-PET/CT assessments		Right liver lobe metastasis SUVmax
D+T	BASELINE	13.11.2018	5,73
D+T	2 WeFU	21.12.2018	6,01
D+T	6 WeFU	08.01.2019	5,91
O+D	2 WeFU	28.01.2019	3,47
O+D	6 WeFU	27.02.2019	4,30
O+D	12 WeFU	10.04.2019	4,17
O+D+T	2 WeFU	20.05.2019	7,13
O+D+T	6 WeFU	21.06.2019	7,58
O+CTX+B (2x)	4 WeFU	22.08.2019	6,26
O+TACE	not done	not done	not done

Supplementary Table 2 (A,B) Treatment strategy, dose regimen and treatment-related adverse events according to Common Terminology Criteria for Adverse Events (CTCAE) Version 5.0. AEs of higher grade were mostly mixed disease- and treatment-related effects. **(A)** P01; **(B)** P04. D+T, dabrafenib+trametinib; O+D(+T), osimertinib+dabrafenib (+trametinib);O(+CTX)+B, osimertinib(+chemotherapy)+bevacizumab; A+C, afatinib+ crizotinib; O+TACE, osimertinib+transarterial chemo- embolization; TTD, time-to-treatment discontinuation; AE, adverse event.

A

Treatment	Start-Stop	Dose regimen	TTD (days)	AEs	Comments
D+T	06.12.2018-18.02.2019	dabrafenib 150mg 1-0-1 trametinib 2mg 0-0-1	74	Hyponatremia Grade II-III; GGT/AP increased Grade I-II; Fever Grade II-III; Nausea Grade I; Lipase/Amylase increased Grade I-II; Fatigue Grade I; Anorexia Grade I; Panniculitis Grade I-II; Anemia Grade I;	
O+D	22.02.2019-21.03.2019	osimertinib 80mg 0-0-1 dabrafenib 150mg 1-0-1	27	Lipase/Aamylase increased Grade II-III; GGT/AP increased Grad I; Anemia Grade I;	Intermittent interruption of O+D due to increase in amylase and lipase (no signs of pancreatitis);
A+C	24.03.2019-28.05.2019	afatinib 40mg 1-0-0 crizotinib 200mg 1-0-1	65	Rash maculo-papular Grade I-II; Diarrhea Grade I; Lipase/ Amylase increased Grade I-II; Nausea Grade I; Edema Grade I; Dry eyes Grade I; Anorexia Grade I; Paronychia Grade I-II; Anemia Grade I;	Intermittent interruption and/or dose reduction of A+C;
O+D+T	05.06.2019-19.03.2020	osimertinib 80mg 0-0-1 dabrafenib 150mg 1-0-1 trametinib 2mg 0-0-1	288	Lipase/Amylase increased Grade II-III; GGT/AP increased Grade I; Hyponatremia Grade II-III; Diarrhea Grade I; Fever Grade I-II; Nausea Grade I; Fatigue Grade I; Anorexia Grade I; Ascites Grade II; Anemia Grade II-III; Edema Grade I;	Intermittent interruption of O+D+T due to increase in amylase and lipase (no symptoms of pancreatitis); Ascites was associated to peritoneal carcinosis and cirrhosis (paracentesis); Tolvaptan treatment was administered for hypo-natremia;
O+B	20.03.2020-12.05.2020	osimertinib 80mg 1-0-0 bevacizumab 15mg/kg Q3W (2x)	53	Ascites Grade II; Fatigue Grade II; Anorexia Grade II; Anemia Grade II; Edema Grade I-II; GGT increased Grade I;	Ascites/Edema/Fatigue/Anorexia were associated with tumor progression, pre-existing cirrhosis and hypo-albuminemia;
A+C	17.05.2020-30.08.2020	afatinib 30mg 1-0-0 crizotinib 250mg 1-0-1, later afatinib was reduced to 20 mg 1-0-0 and crizotinib to 200mg 1-0-1	105	Ascites Grade II-III; Fatigue Grade II; Anorexia Grade II; Anemia Grade II; Edema Grade I-II; Rash Grade I-II; GGT/AP increased Grade I-II; Diarrhea Grade I-II; Nausea Grade II;	Intermittent interruption and/or dose reduction of afatinib and crizotinib due to rash, edema, diarrhea or nausea;
O+D+T	02.09.2020-ongoing	osimertinib 80mg 0-0-1 dabrafenib 150mg 1-0-1 trametinib 2mg 0-0-1	na	Ascites Grade II-III; Fatigue Grade II; Anorexia Grade II; Anemia Grade II; Edema Grade I-II; GGT/AP increased Grade I-II; Diarrhea Grade I-II; Nausea Grade II;	

B

Treatment	Start-Stop	Dose regimen	TTD (days)	AEs	Comments
D+T	06.12.2018-13.01.2019	dabrafenib 150mg 1-0-1 trametinib 2mg 0-0-1	38	Nausea Grade I; Vomiting Grad I; GGT/AP increased Grade I; Fatigue Grade I; Fever Grade I; Dry mouth Grade I; Myalgia Grade I-II;	Increase in AP/GGT were associated with liver metastases;
O+D	14.01.2019-17.04.2019	osimertinib 80mg 0-0-1 dabrafenib 150mg 1-0-1	93	Nausea Grade I; Fatigue Grade I; GOT/GPT increased Grade I; GGT/AP increased Grade II-III; Cough Grade I; Lipase increased Grade I; Myalgia Grade I-II; Anorexia Grade I;	Increase in GOT/GPT can be related to treatment; Increase in AP/GGT can be associated with liver metastases; Cough was possibly associated with respiratory infection and resolved within a few days;
O+D+T	18.04.2019-04.07.2019	osimertinib 80mg 0-0-1 dabrafenib 150mg 1-0-1 trametinib 2mg 0-0-1, from 30.04.2019 trametinib was reduced to 1mg 0-0-1	77	Myalgia Grade II; Anorexia Grade I; GGT/AP increased Grade II; Fatigue Grade I; Fever Grade I;	Dose of trametinib was reduced which improved myalgia and fatigue symptoms;
O+CTX+B	05.07.2019-18.09.2019	osimertinib 80mg 1-0-0 carboplatin AUC 6 and pemetrexed 500 mg/m^2 (2x cycles; 2nd cycle: AUC 4 and $250/m^2$) bevacizumab 15 mg/kg Q3W (2x cycles)	75	Fatigue Grade II; Anorexia II-III; GGT/AP increased Grade II-III; Fatigue Grade I;	Second cycle of chemo-therapy was dose reduced due to clinical deterioration of the patient.
O+TACE	19.09.2019-Nk.09.2019	osimertinib 80mg 1-0-0	na	Ascites Grade II-III; Fatigue Grade II; Anorexia Grade II; Anemia Grade II; Edema Grade I-II; GGT/AP increased Grade I-II; Diarrhea Grade I-II; Nausea Grade II;	

Supplementary Table 3 Used Primers

Sequence	Purpose
CTAAGCCTCCGCCTCCTC	pBabe seq f
GACTAATTGAGATGCATG	pBabe seq r
TCCGCTGTCAAACATGTGGT	seq PBABE V600E inside Braf-casette
TCGTGGTGATGGAGGATCAAC	BRAF qPCR f
TCATCACTCGAGTCCCGTCT	BRAF qPCR r
CAGGTGGTGTTGGGAAAAGC	NRAS qPCR f
TCAACACCCTGTCTGGTCTT	NRAS qPCR r

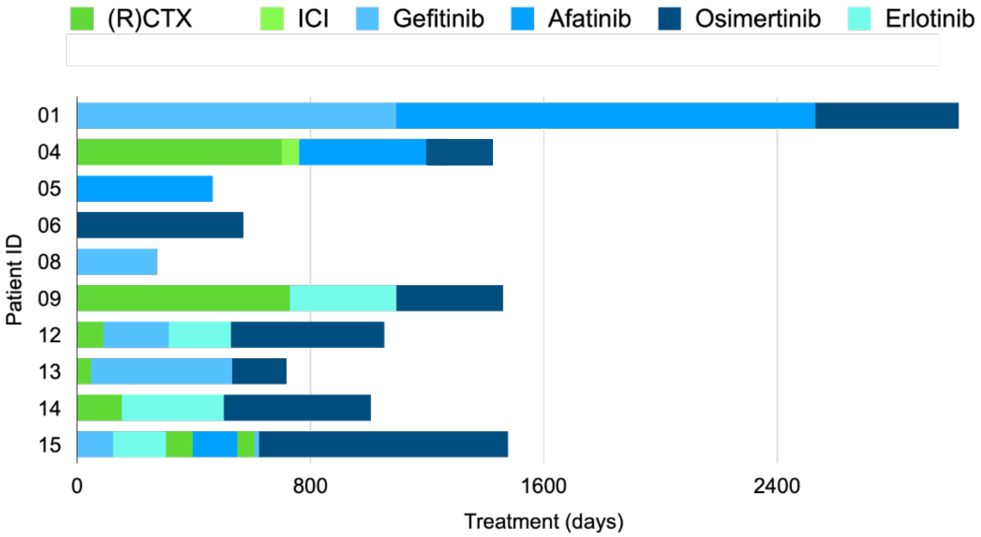

Supplementary Figure 1. Treatment history before the detection of acquired *BRAF* mutations in 10 patients evaluable for treatment history.

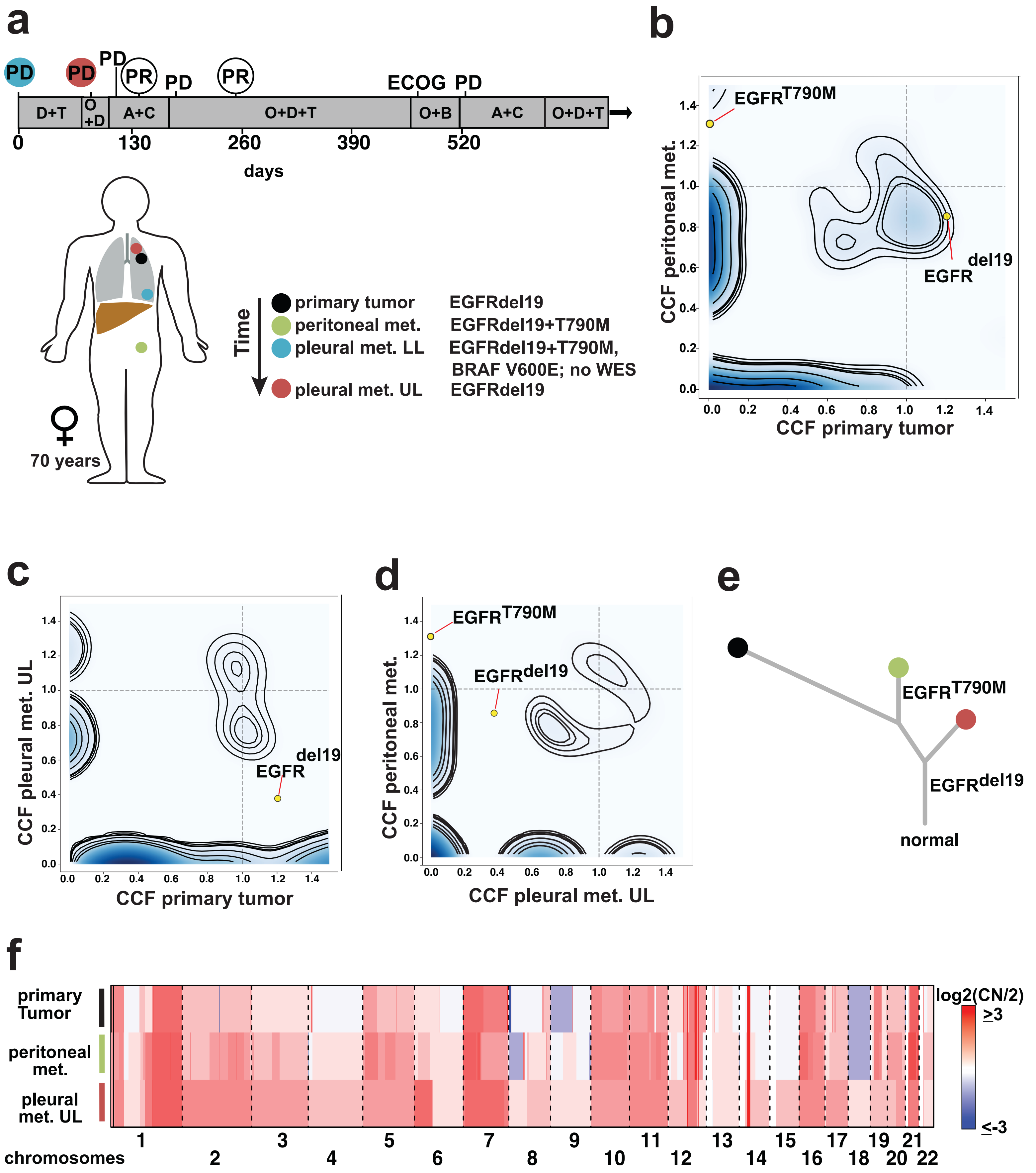

Supplementary Figure 2. (**a**) Overview of the biopsies and key molecular findings by targeted NGS for patient 01 and flow chart summarizing lines of therapy approaches over time after the acquisition of *BRAF*^{V600E} mutation following osimertinib. (**b-d**) Pairwise clustering of WES-derived mutations based on their CCFs between pairs of tumor biopsies. Large clusters of private mutations indicate a high degree of genetic dissimilarity between biopsies. Candidate mutations in *EGFR* are highlighted. (**e**) Visualization of genetic distances between normal tissue and longitudinal biopsies in a phylogenetic tree. Branching indicates that the metastases and the primary tumor derived from a shared common ancestor. (**f**) Profiles of purity and ploidy corrected copy number (CN) in the metastases. (red = CN gain, blue = CN loss). *EGFR*, epidermal growth factor receptor; *BRAF*, B-rapidly accelerated fibrosarcoma; WES, whole-exome sequencing; NGS, next-generation sequencing; PD, progressive disease; PR, partial response; D+T, dabrafenib+trametinib; O+D(+T), osimertinib+dabrafenib(+trametinib); A+C, afatinib+crizotinib; O+B, osimertinib+bevacizumab; ECOG, Eastern Cooperative Oncology Group; CCF, cancer cell fraction; LL, lower lobe; UL, upper lobe.

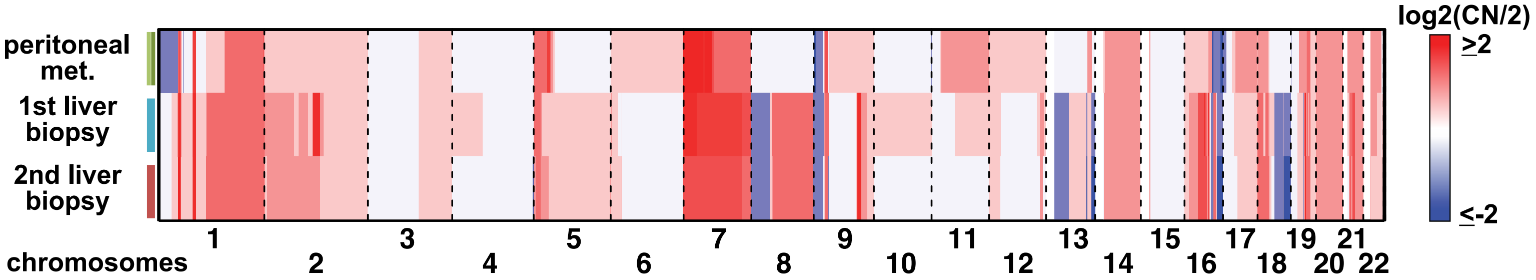

Supplementary Figure 3. Profiles of purity and ploidy corrected copy number (CN) in the metastases (red = CN gain, blue = CN loss) for patient P04.

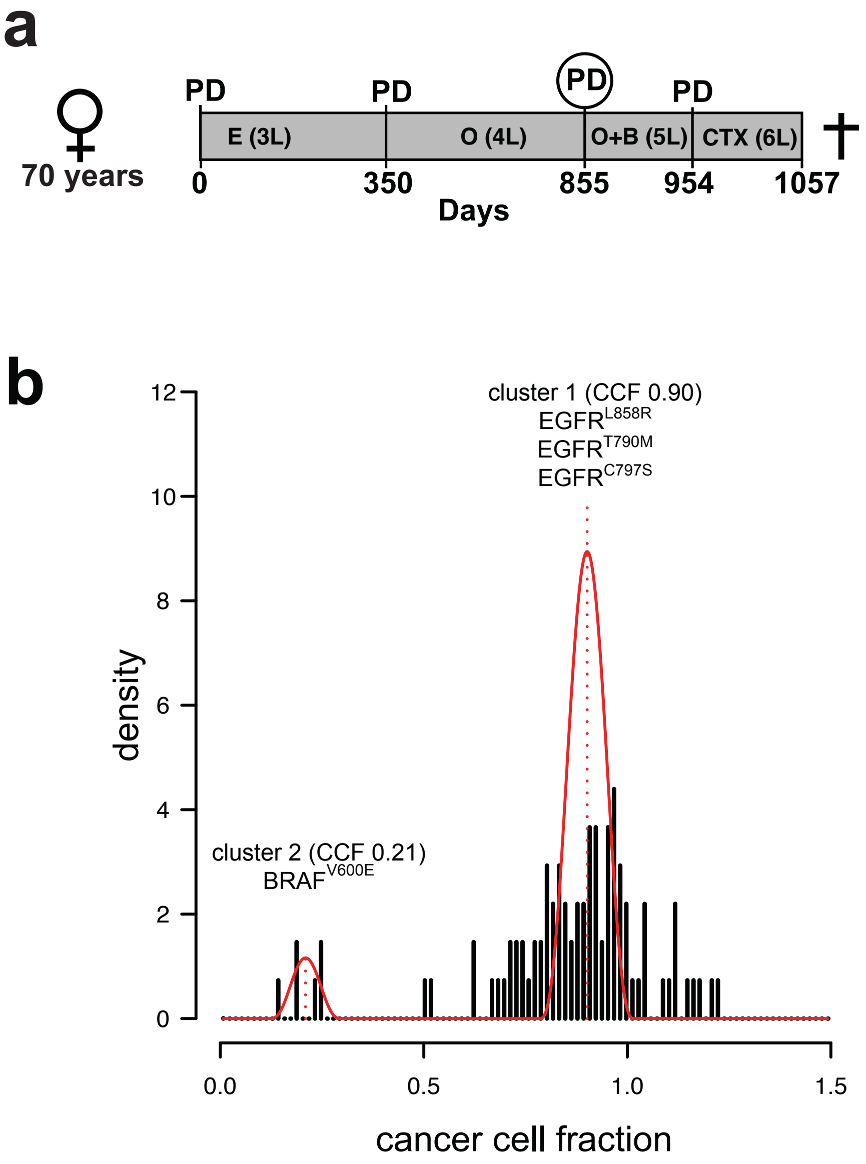

Supplementary Figure 4. (**a**) Overview of the treatment lines for patient P14 after erlotinib was started. Biopsy for WES obtained at the time of progression after osimertinib treatment was taken at day 855. (**b**) WES-based clonality analysis of the biopsy displayed two mutation clusters with corresponding cancer cell fractions (CCF). Relevant mutations are indicated above the corresponding clusters. *EGFR*, epidermal growth factor receptor; *BRAF*, B-rapidly accelerated fibrosarcoma; WES, whole-exome sequencing; PD, progressive disease; E, erlotinib; O, osimertinib; O+B, osimertinib+bevacizumab; CTX, chemotherapy. L, line of therapy.

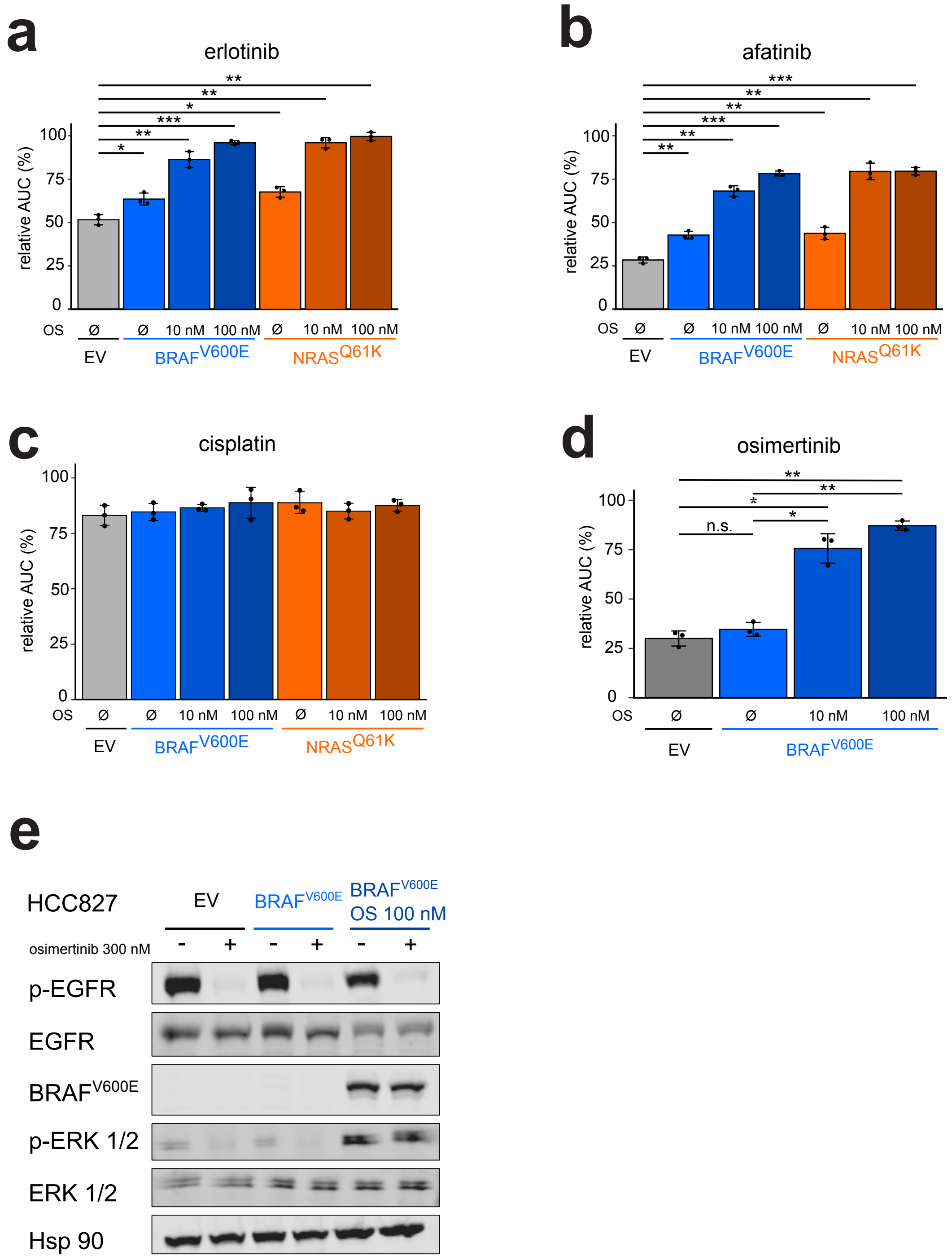

Supplementary Figure 5. (**a-c**) Viability assay of PC9 derived cell lines, treated for 72 hours with (**a**) erlotinib, (**b**) afatinib or (**c**) cisplatin. (**d**) Viability assay of HCC827 derived cell lines treated with osimertinib (72h) are shown. (**e**) Immunoblotting of HCC827 cells expressing the annotated constructs, treated with (+) or without (-) osimertinib (48h) and Hsp90 is used as loading control. The relative area under the curve (AUC) in % compared to a theoretical non-responding AUC. Error bars indicate mean ± SD. Two-tailed paired t-tests, ***$p < 0.001$, **$p < 0.01$, *$p < 0.05$.

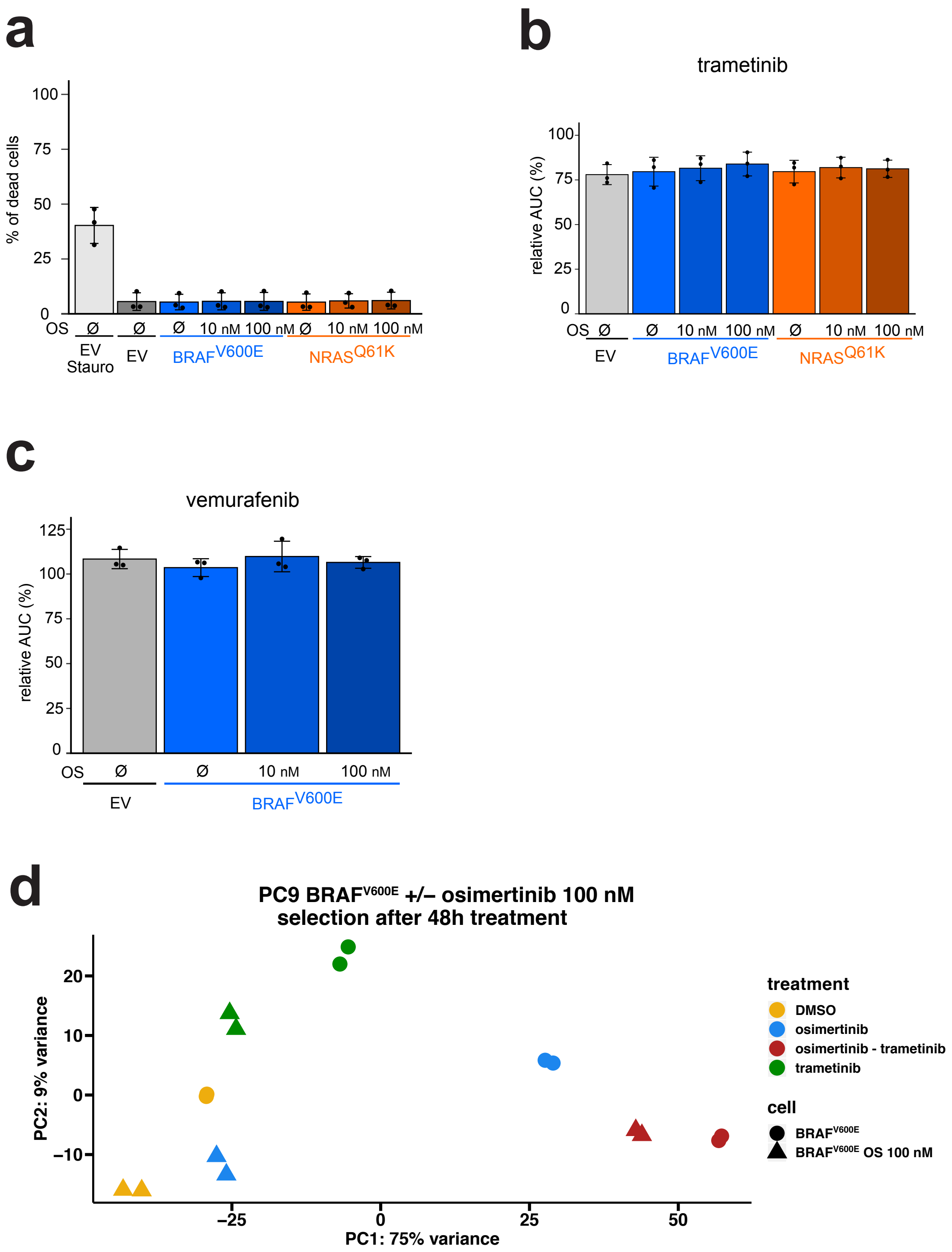

Supplementary Figure 6. (**a**) Percentage of dead cells measured by flow cytometry. Staurosporine control treated for 24 hours. (**b,c**) Viability assay of PC9 (EV) derived cell lines, treated for 72 hours with (**b**) trametinib or (**c**) vemurafenib. (**d**) Principal component analysis of 3’UTR-RNA-seq-samples in duplicates. The relative area under the curve (AUC) in % compared to a theoretical non-responding AUC. Error bars indicate mean ± SD. Two-tailed paired t-tests, *** $p < 0.001$, **$p < 0.01$, *$p < 0.05$.

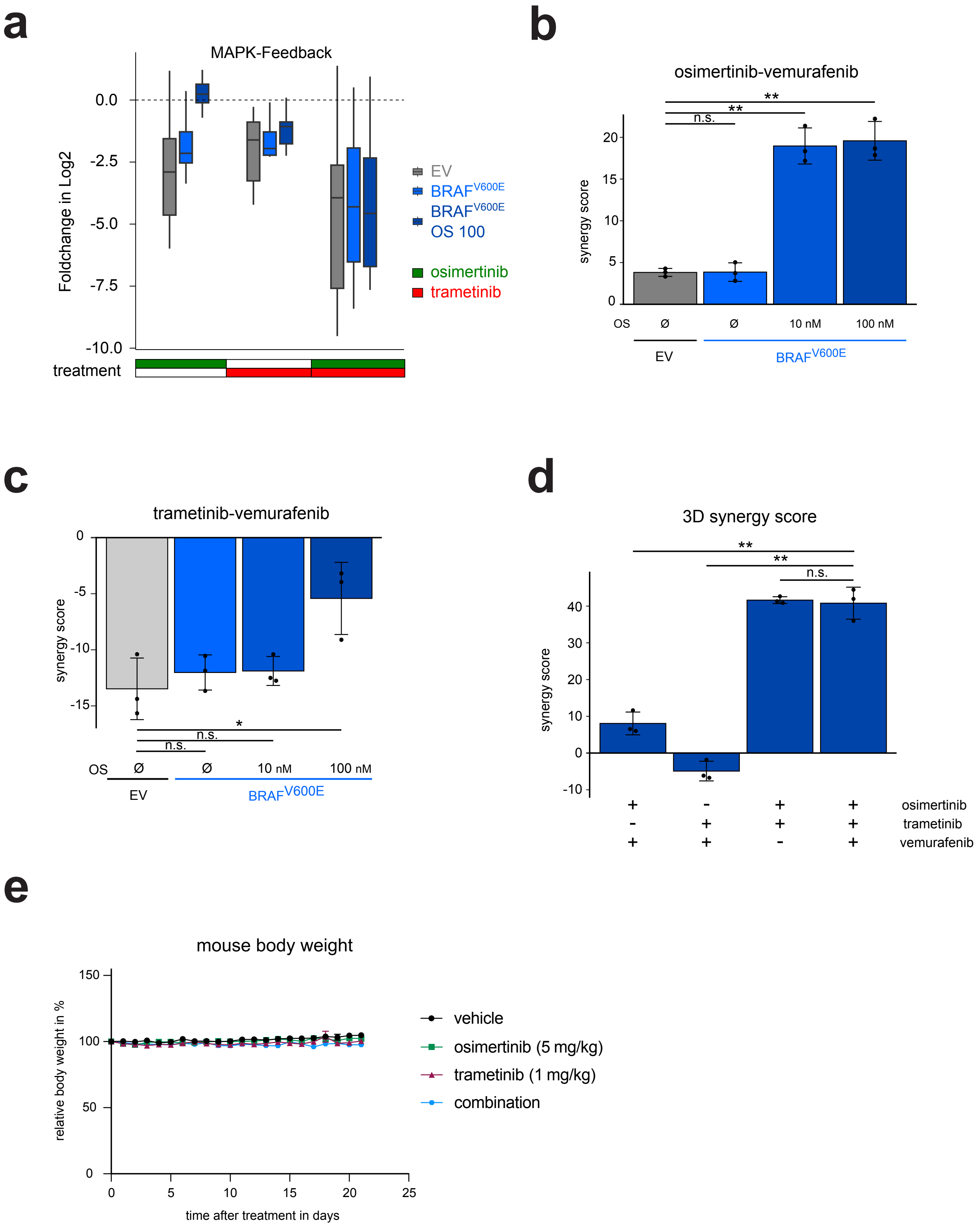

Supplementary Figure 7. (**a**) RNA-seq based log2 fold-changes of negative MAPK feedback genes (see methods) of PC9 derived cell lines after 48h treatment with indicated inhibitors compared to their respective DMSO controls. (**b**) Synergy screen of osimertinib and vemurafenib combination treatment in PC9 derived cell lines for 72 hours. (**c**) Synergy screen of trametinib and vemurafenib combination treatment in PC9 derived cell lines for 72 hours. (**d**) 3D Synergy screen of osimertinib, trametinib and vemurafenib combination treatment in PC9 pBABE BRAFV600E OS 100 cells for 72 hours. (**e**) Relative body weight of all mice in trial (see methods) in % compared to day 0. Error bars indicate mean ± SD. Two-tailed paired t-tests, *** $p < 0.001$, ** $p < 0.01$, * $p < 0.05$.

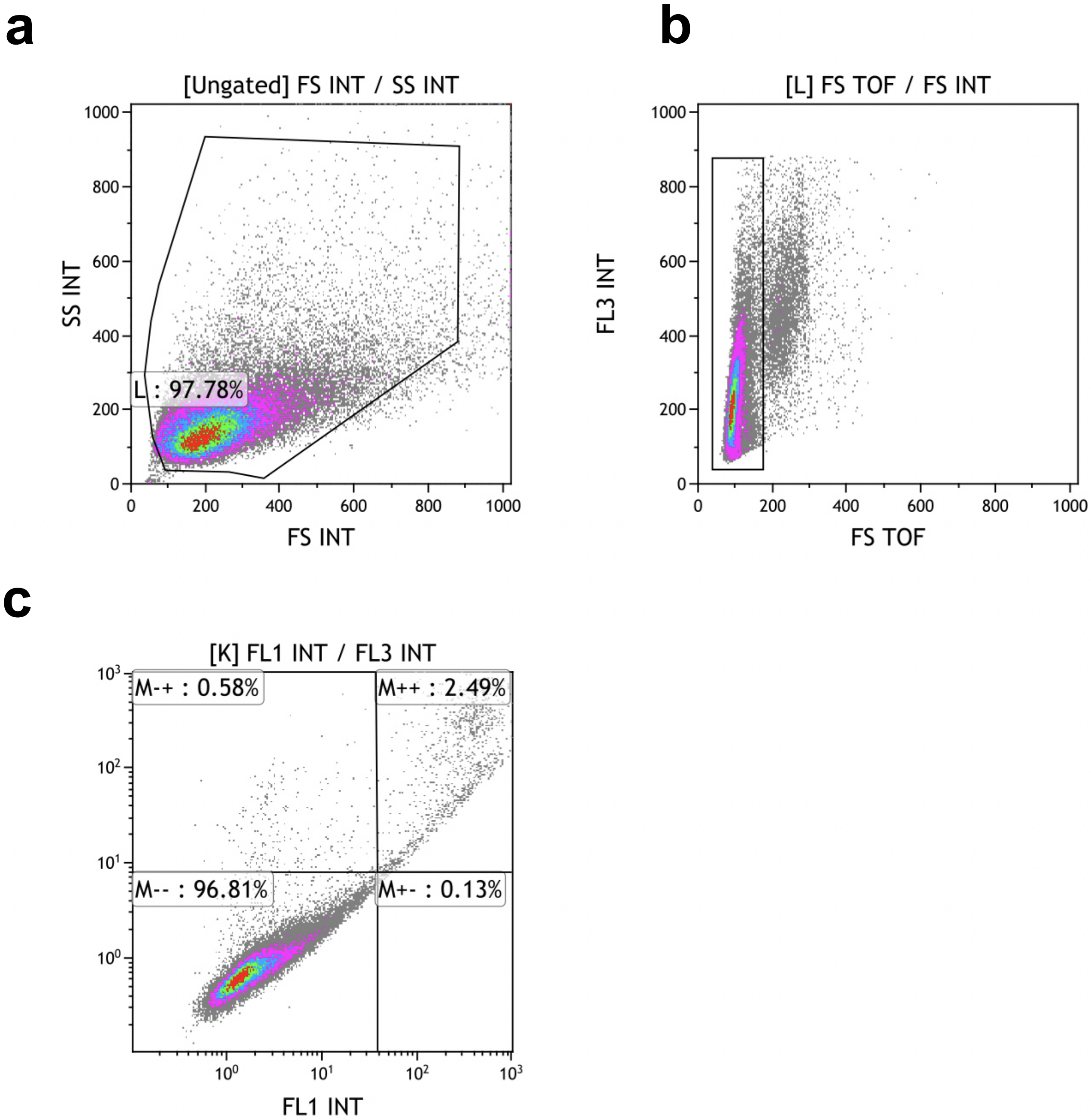

Supplementary Figure 8. Flow cytometry gating strategy. Apoptosis assay using flow cytometry after staining with annexin V-FITC/propidium iodide (PI). (**a**) Total cells were first gated on a forward scatter (FS)/side scatter (SS) for total counted events. (**b**) Cells were gated on a FS area versus FS width density plot to remove doublet cells. (**c**) Representative scatter plots of PI (y-axis) vs. annexin V (x-axis).

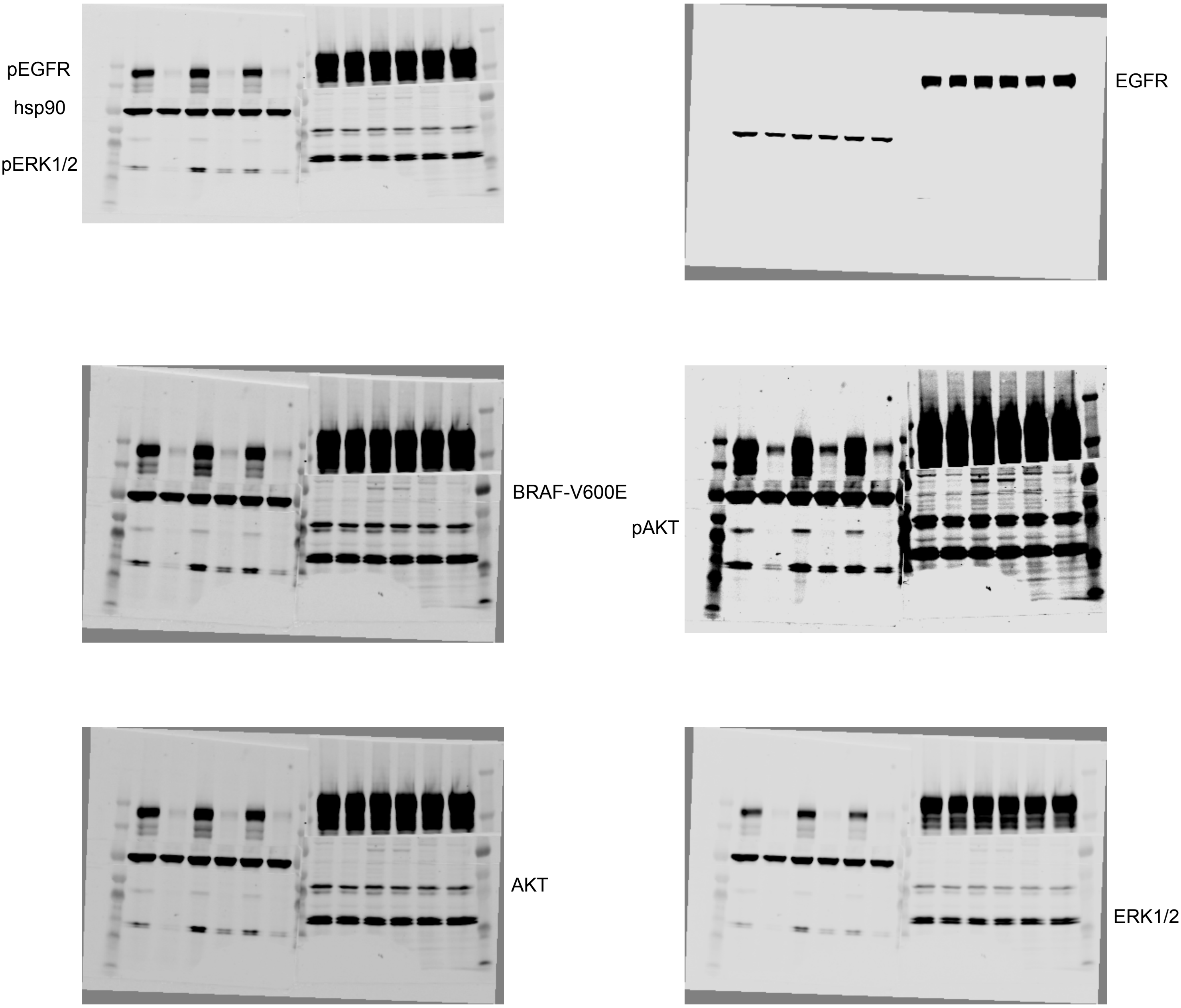

Supplementary Figure 9. Uncropped Blots of Figure 2a

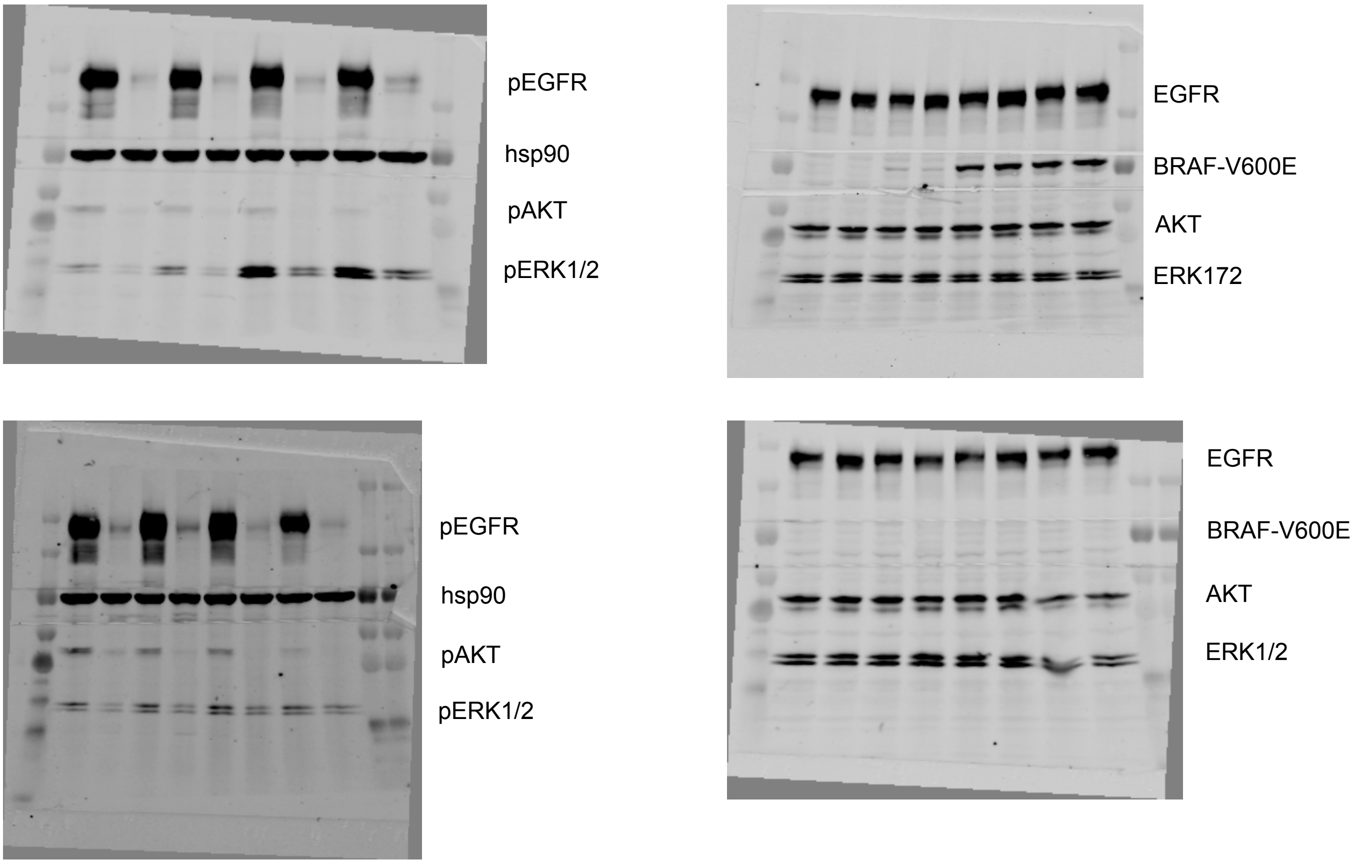

Supplementary Figure 10. Uncropped blots of Figure 2g

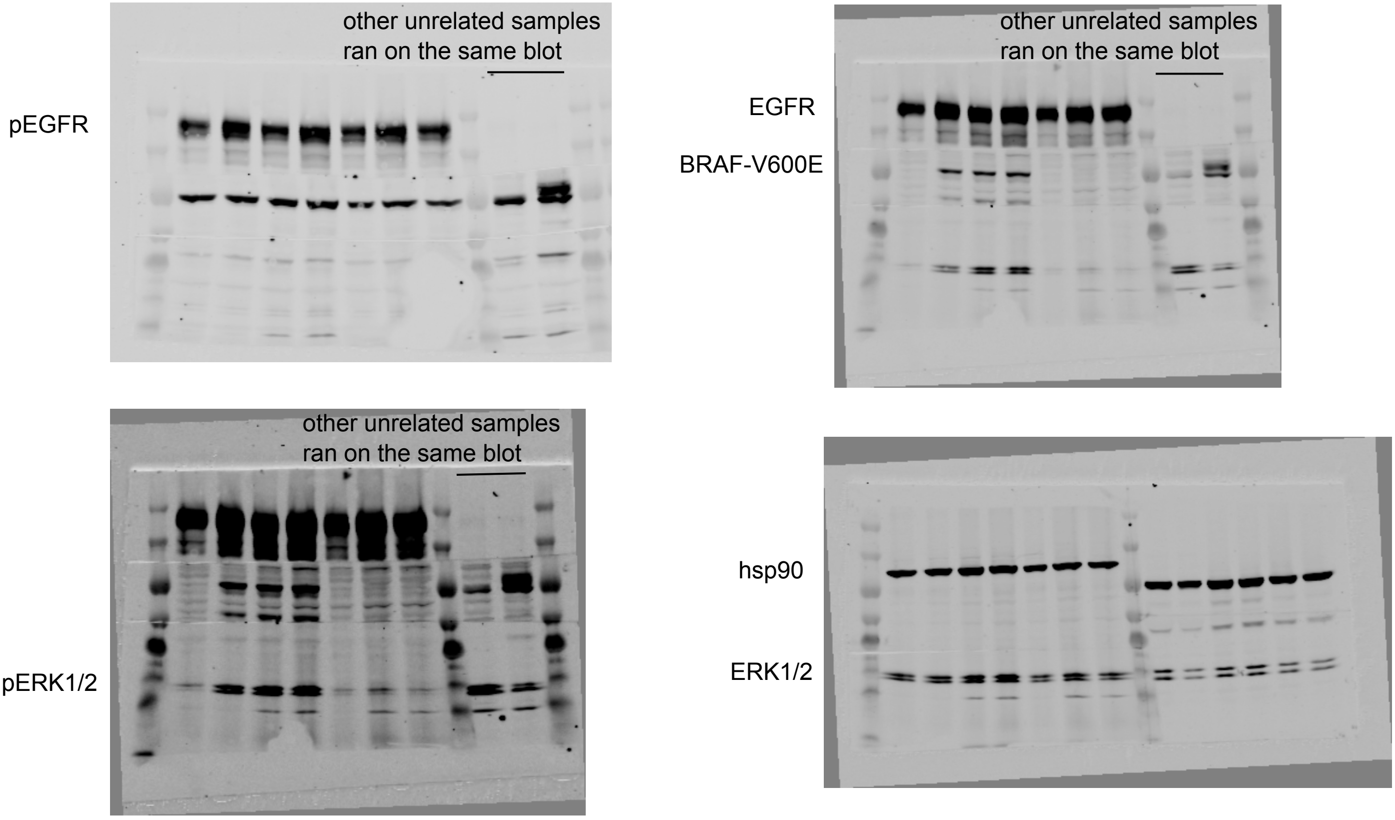

Supplementary Figure 11. Uncropped blots of Figure 3b

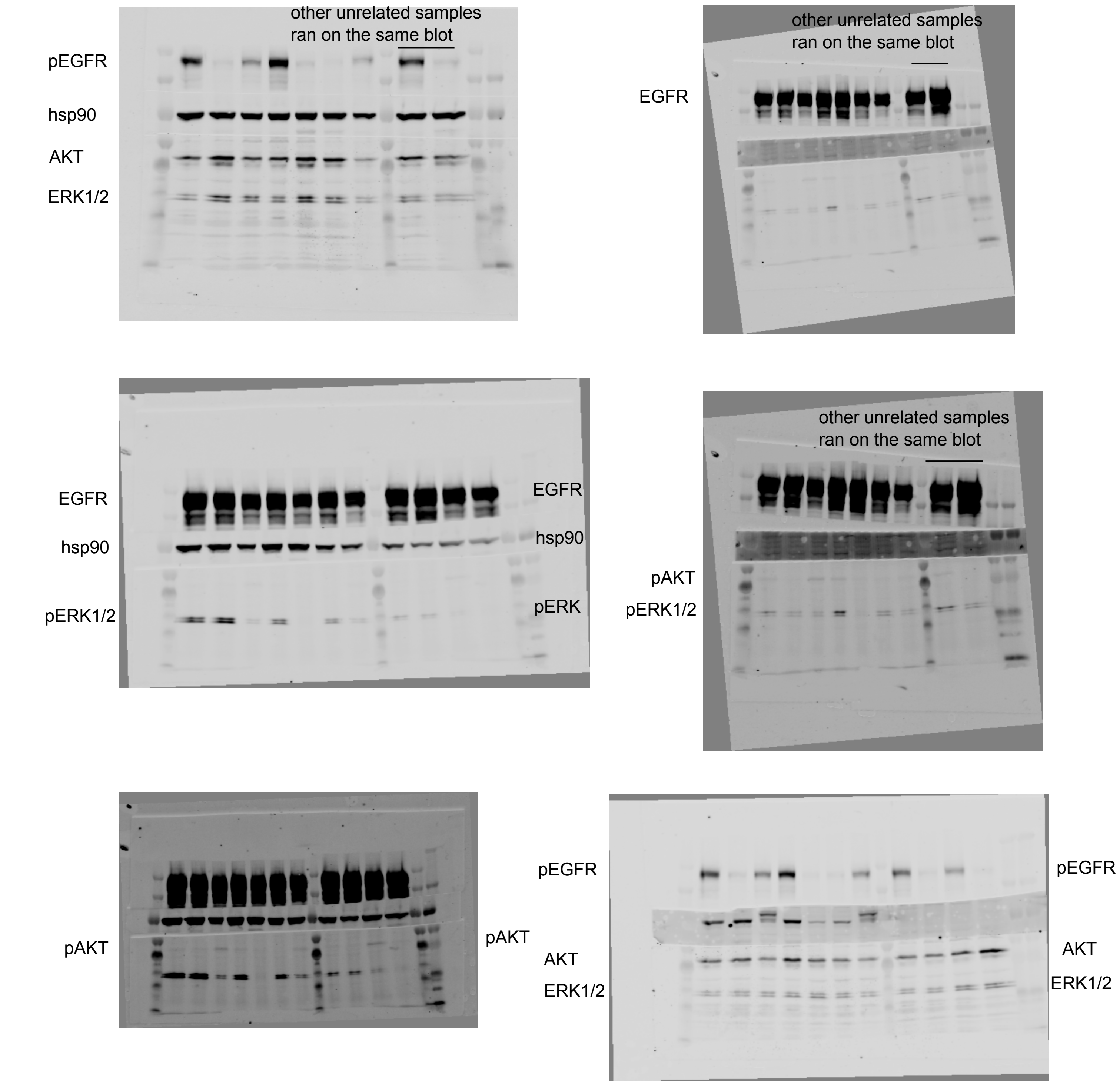

Supplementary Figure 12. Uncropped blots of Figure 3g

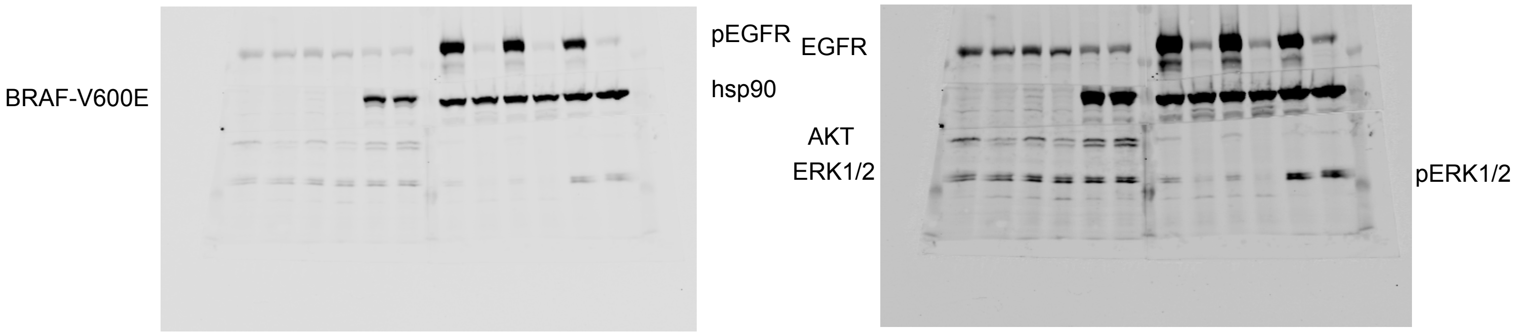

Supplementary Figure 13. Uncropped blots of Supplementary Figure 5e
