# Supplementary material for: Clonal dynamics of BRAF-driven drug resistance in EGFR-mutant lung cancer
Source: NPJ Precis Oncol. 2021 Dec 17;5:102. doi: 10.1038/s41698-021-00241-9 (PMC8683498; doi:10.1038/s41698-021-00241-9)
Supplement: Supplementary file 2 — Reporting Summary [file 41698_2021_241_MOESM2_ESM.pdf]

nature portfolio

Corresponding author(s): Prof. Dr. Martin L. Sos
PD Dr. Matthias Scheffler
Dr. Johannes Brägelmann

Last updated by author(s): Oct 22, 2021

Reporting Summary

Nature Portfolio wishes to improve the reproducibility of the work that we publish. This form provides structure for consistency and transparency in reporting. For further information on Nature Portfolio policies, see our Editorial Policies and the Editorial Policy Checklist.

Statistics

For all statistical analyses, confirm that the following items are present in the figure legend, table legend, main text, or Methods section.

n/a	Confirmed	
☐	☒	The exact sample size (n) for each experimental group/condition, given as a discrete number and unit of measurement
☐	☒	A statement on whether measurements were taken from distinct samples or whether the same sample was measured repeatedly
☐	☒	The statistical test(s) used AND whether they are one- or two-sided<br>*Only common tests should be described solely by name; describe more complex techniques in the Methods section.*
☒	☐	A description of all covariates tested
☐	☒	A description of any assumptions or corrections, such as tests of normality and adjustment for multiple comparisons
☐	☒	A full description of the statistical parameters including central tendency (e.g. means) or other basic estimates (e.g. regression coefficient) AND variation (e.g. standard deviation) or associated estimates of uncertainty (e.g. confidence intervals)
☐	☒	For null hypothesis testing, the test statistic (e.g. F, t, r) with confidence intervals, effect sizes, degrees of freedom and P value noted<br>*Give P values as exact values whenever suitable.*
☒	☐	For Bayesian analysis, information on the choice of priors and Markov chain Monte Carlo settings
☒	☐	For hierarchical and complex designs, identification of the appropriate level for tests and full reporting of outcomes
☒	☐	Estimates of effect sizes (e.g. Cohen's d, Pearson's r), indicating how they were calculated

Our web collection on statistics for biologists contains articles on many of the points above.

Software and code

Policy information about availability of computer code

Data collection	Data were acquired with ImageStudio v.5.2.5 (LI-COR Bioscience), Tecan i-control v.3.9.1 (Tecan, Austria).
Data analysis	Data were analyzed with GraphPad Prism v6.0, Integrated Genome Viewer v2.3.68, RSEM v1.3.1, STAR v2.7.0, samtools v1.3, FACS Kaluza software, Geneious Prime v2019.0.4, and the statistical programming environment R v3.5.0.

For manuscripts utilizing custom algorithms or software that are central to the research but not yet described in published literature, software must be made available to editors and reviewers. We strongly encourage code deposition in a community repository (e.g. GitHub). See the Nature Portfolio guidelines for submitting code & software for further information.

Data

Policy information about availability of data

All manuscripts must include a data availability statement. This statement should provide the following information, where applicable:
- Accession codes, unique identifiers, or web links for publicly available datasets
- A description of any restrictions on data availability
- For clinical datasets or third party data, please ensure that the statement adheres to our policy

The source data that support the findings of this work are available from the corresponding authors upon reasonable request. Additional data regarding e.g. material, methods, and results are available in the Supplementary Material file.

Field-specific reporting

Please select the one below that is the best fit for your research. If you are not sure, read the appropriate sections before making your selection.

- [x] Life sciences
- [ ] Behavioural & social sciences
- [ ] Ecological, evolutionary & environmental sciences

For a reference copy of the document with all sections, see nature.com/documents/nr-reporting-summary-flat.pdf

Life sciences study design

All studies must disclose on these points even when the disclosure is negative.

Sample size

Clinical design, patients: We compiled a cohort of 15 patients with lung adenocarcinoma and activating EGFR mutations that harbored co-occurring BRAF mutations with and without prior anti-EGFR treatment. Patients were identified within the Network Genomic Medicine (NGM) Lung Cancer in Cologne, Germany, Institute Gustave Roussy in Paris, France, and Cantonal Hospital of Lucerne, Switzerland. Treatment, genetic findings, and survival of these patients were evaluated.

Experimental design: Sample sizes were not determined a priori. Generally accepted sample sizes were used, with reproducable differences between conditions indicating that the chosen sample sizes were sufficient.

Data exclusions

No exclusion data to disclose.

Replication

For in vitro experiments at least three independent biological replicates were performed with technical replicates per experiment whenever feasible (e.g. viability assays). Technical replicates were averaged first and then processed further. All figures thus show averages and variability calculated from at least three independent experiments. All experimental replicates were included in the final analyses and successfully validated the experimental findings within the reported degree of variability.

Randomization

Clinical design, patients: No randomization. We compiled a cohort of 15 patients with lung adenocarcinoma and activating EGFR mutations that harbored co-occurring BRAF mutations with and without prior anti-EGFR treatment. Patients were identified within the Network Genomic Medicine (NGM) Lung Cancer in Cologne, Germany, Institute Gustave Roussy in Paris, France, and Cantonal Hospital of Lucerne, Switzerland. Treatment, genetic findings, and survival of these patients were evaluated.

Experimental design: For in vivo analysis, tumour-bearing mice were randomized based on tumour size before treatment to obtain an equal mean tumour size and similar distribution of individual sizes at treatment start.

Blinding

The investigators including molecular pathologists, radiologists, bioinformatics except for the clinicians were blinded to treatment and patient characteristics (e.g. age, gender) during data collection and analysis until the study was completed.

Reporting for specific materials, systems and methods

We require information from authors about some types of materials, experimental systems and methods used in many studies. Here, indicate whether each material, system or method listed is relevant to your study. If you are not sure if a list item applies to your research, read the appropriate section before selecting a response.

Materials & experimental systems

n/a	Involved in the study	
[ ]	[x]	Antibodies
[ ]	[x]	Eukaryotic cell lines
[x]	[ ]	Palaeontology and archaeology
[ ]	[x]	Animals and other organisms
[ ]	[x]	Human research participants
[ ]	[x]	Clinical data
[x]	[ ]	Dual use research of concern

Methods

n/a	Involved in the study	
[x]	[ ]	ChIP-seq
[x]	[ ]	Flow cytometry
[x]	[ ]	MRI-based neuroimaging

Antibodies

Antibodies used

EGFR (CS-4267, Cell Signaling), p-EGFR (CS-3777, Cell signaling), BRAF-V600E (E-19290, Spring Bioscience), BRAF (SC-5284, Santa Cruz), ERK (CS-9102, Cell Signaling), p-ERK (CS-4370, Cell Signaling), Akt (CS-2920, Cell Signaling), p-Akt (CS-9271, Cell Signaling) and Hsp90 (CS-4877, Cell Signaling)

Validation

All antibodies are commercially available and have been validated by the manufacturer.

Eukaryotic cell lines

Policy information about cell lines

Cell line source(s)	Cell lines were obtained from ATCC.
Authentication	Cell lines were authenticated by STR profiling at the Institute of Forensic Medicine of the University Hospital of Cologne.
Mycoplasma contamination	All cell lines were tested to be Mycoplasma negative at regular intervals.
Commonly misidentified lines (See ICLAC register)	Commonly misidentified cell lines were not used.

Animals and other organisms

Policy information about studies involving animals; ARRIVE guidelines recommended for reporting animal research

Laboratory animals	8-12 weeks-old female nude mice (RJ:NMRI-FOXN1 NU, Janvier Labs)
Wild animals	NA
Field-collected samples	NA
Ethics oversight	All in vivo experiments were approved by the local authorities and the animal protection commitee.

Note that full information on the approval of the study protocol must also be provided in the manuscript.

Human research participants

Policy information about studies involving human research participants

Population characteristics	Please refer to the manuscript and supplementary material for details. Clinicopathological characteristics for the study cohort are summarized e.g. in Table 1 of the manuscript.
Recruitment	We compiled a cohort of 15 patients with lung adenocarcinoma and activating EGFR mutations that harbored co-occurring BRAF mutations with and without prior anti-EGFR treatment. Patients were identified within the Network Genomic Medicine (NGM) Lung Cancer in Cologne, Germany, Institute Gustave Roussy in Paris, France, and Cantonal Hospital of Lucerne, Switzerland. Treatment, genetic findings, and survival of these patients were evaluated. Please refer to the manuscript and supplementary material for details
Ethics oversight	Patients were identified within the Network Genomic Medicine (NGM) Lung Cancer in Cologne, Germany, Institute Gustave Roussy in Paris, France, and Cantonal Hospital of Lucerne, Switzerland. The study was conducted in concordance with local ethical guidelines and was reviewed by the institutional ethics committee.

Note that full information on the approval of the study protocol must also be provided in the manuscript.

Clinical data

Policy information about clinical studies

All manuscripts should comply with the ICMJEguidelines for publication of clinical research and a completedCONSORT checklist must be included with all submissions.

Clinical trial registration	No clinical trial registration number.
Study protocol	No clinical trial registration. Please refer to the manuscript and supplementary material for details on the study design and conduct.
Data collection	Patients with lung adenocarcinoma and activating EGFR mutations that harbored co-occurring BRAF mutations with and without prior anti-EGFR treatment were identified within the Network Genomic Medicine (NGM) Lung Cancer in Cologne, Germany, Institute Gustave Roussy in Paris, France, and Cantonal Hospital of Lucerne, Switzerland. Clinicopathological characteristics, treatment outcome, genetic findings, and survival of these patients were evaluated. Please refer to the manuscript and supplementary material for details.
Outcomes	Patients with lung adenocarcinoma and activating EGFR mutations that harbored co-occurring BRAF mutations with and without prior anti-EGFR treatment were evaluated for clinicopathological characteristics, treatment outcome, genetic findings, and survival.
